# An improved *Nicotiana benthamiana* bioproduction chassis provides novel insights into nicotine biosynthesis

**DOI:** 10.1111/nph.19141

**Published:** 2023-07-24

**Authors:** Katharina Vollheyde, Quentin M. Dudley, Ting Yang, Mehmet T. Oz, Davide Mancinotti, Mariano Olivera Fedi, Darren Heavens, Gareth Linsmith, Monika Chhetry, Mark A. Smedley, Wendy A. Harwood, David Swarbreck, Fernando Geu‐Flores, Nicola J. Patron

**Affiliations:** ^1^ Department of Plant and Environmental Sciences University of Copenhagen 1871 Frederiksberg Copenhagen Denmark; ^2^ Earlham Institute, Norwich Research Park Norwich Norfolk NR4 7UZ UK; ^3^ John Innes Centre, Norwich Research Park Norwich Norfolk NR4 7UH UK

**Keywords:** alkaloid biosynthesis, gene editing, metabolic engineering, *Nicotiana benthamiana*, nicotine

## Abstract

- The model plant *Nicotiana benthamiana* is an increasingly attractive organism for the production of high‐value, biologically active molecules. However, *N. benthamiana* accumulates high levels of pyridine alkaloids, in particular nicotine, which complicates the downstream purification processes.
- Here, we report a new assembly of the *N. benthamiana* genome as well as the generation of low‐nicotine lines by CRISPR/Cas9‐based inactivation of berberine bridge enzyme‐like proteins (BBLs). Triple as well as quintuple mutants accumulated three to four times less nicotine than the respective control lines.
- The availability of lines without functional BBLs allowed us to probe their catalytic role in nicotine biosynthesis, which has remained obscure. Notably, chiral analysis revealed that the enantiomeric purity of nicotine was fully lost in the quintuple mutants. In addition, precursor feeding experiments showed that these mutants cannot facilitate the specific loss of C6 hydrogen that characterizes natural nicotine biosynthesis.
- Our work delivers an improved *N. benthamiana* chassis for bioproduction and uncovers the crucial role of BBLs in the stereoselectivity of nicotine biosynthesis.

## Introduction

*Nicotiana benthamiana* is a widely used model plant native to Australia (Goodin *et al*., 2008; Bally *et al*., 2018). Following early observations of hypersusceptibility to viruses, *N. benthamiana* became valued for studying plant–pathogen interactions, and seeds were shared among the global plant science community (Bally *et al*., 2015). The hypersusceptibility trait was eventually linked to a disruptive mutation in *RNA‐dependent RNA polymerase 1* (*Rdr1*) present in ecotypes from arid regions (Bally *et al*., 2015). The mutation trades viral defense in favor of early vigor and thus confers an advantage in climates with low and unpredictable rainfall, where lifecycles must be completed in limited time (Bally *et al*., 2015; Karasov *et al*., 2017). Young *N. benthamiana* plants are also highly susceptible to *Agrobacterium tumefaciens*, a trait which has been exploited for transient gene expression in leaf tissues using a technique known as agroinfiltration (Q. Chen *et al*., 2013). In the last decade, facilities for the agroinfiltration of large numbers of plants have been constructed, thus enabling commercial‐scale protein production (D'Aoust *et al*., 2010; Q. Chen *et al*., 2013; Lomonossoff & D'Aoust, 2016). Notably, *N. benthamiana* leaves can be agroinfiltrated using a complex mixture of *Agrobacterium* strains, leading to the transient co‐expression of a multiplicity of genes. This has been exploited by the plant natural product community, who adopted it as the gold standard for enzyme discovery in the context of pathway elucidation (Geu‐Flores *et al*., 2009; Gnanasekaran *et al*., 2015; Lau & Sattely, 2015; Reed *et al*., 2017; Davis *et al*., 2020; Stephenson *et al*., 2020; Dudley *et al*., 2022a). As more and more natural product pathways are elucidated this way, the facilities constructed for industrial protein production are likely to be repurposed for the production of high‐value plant natural products such as terpenoids (Forman *et al*., 2022) or alkaloids (Nett & Sattely, 2021).

*Nicotiana benthamiana* is in the *Suaveolentes* section of the *Nicotiana* genus, all of which are allopolyploids thought to be descendants of a single hybridization event (Chase *et al*., 2003; Clarkson *et al*., 2004; Kelly *et al*., 2010; Bally *et al*., 2015). The basic haploid chromosome number of *Nicotiana* is *n* = 12, and it is assumed that the ancestral tetraploid had 24 pairs of chromosomes. However, *N. benthamiana* has 19 pairs, the result of diploidization, and chromosomal rearrangements (Chase *et al*., 2003; Kelly *et al*., 2010). Due to its complexity, early *N. benthamiana* genome drafts constructed from short reads were fragmented (Bombarely *et al*., 2012; Naim *et al*., 2012). New sequencing technologies that generate long‐reads aid the production of highly contiguous reference genomes, which improve the identification of large gene families, especially those that tend to occur in polymorphic gene clusters. They also enable studies of genome structure, synteny, and chromosomal rearrangements (Pucker *et al*., 2022). During the preparation of this manuscript, a new draft *N. benthamiana* genome, assembled from Pacific BioScience Highly Accurate Long Read Sequencing reads (PacBio HiFi), was reported (Kurotani *et al*., 2023).

While *N. benthamiana* is a promising chassis for biomanufacturing, the yield and purity of protein products can be hampered by unintended proteolysis (Grosse‐Holz *et al*., 2018). Similarly, small molecules can be further metabolized by oxidoreductases, hydrolases, and transferases that participate in *N. benthamiana*'s own metabolism or xenobiotic detoxification systems (van Herpen *et al*., 2010; Liu *et al*., 2011; Brückner & Tissier, 2013; Miettinen *et al*., 2014; Dong *et al*., 2016; Dudley *et al*., 2022a). In addition, like other *Nicotiana* species, *N. benthamiana* accumulates high amounts of nicotine and related pyridine alkaloids. Nicotine is the most abundant of these, with levels varying from 2.9 to 14.28 mg g^−1^ dry leaf weight (DW) (Sisson & Severson, 1990). This is of concern when producing therapeutic or dietary proteins, as nicotine is highly neuroactive and also potentially toxic in large amounts. In the production of thaumatin II, a small protein from the katemfe fruit used as sweetener, a chromatographic purification step was able to lower the levels of pyridine alkaloids down to levels encountered naturally in other Solanaceae vegetables such as tomato or pepper (*GRAS Notice 910*, 2020). We predict that the presence of nicotine will be more problematic for the industrial production of small molecules, in particular, other alkaloids with similar physio‐chemical properties.

**Fig. 1:**
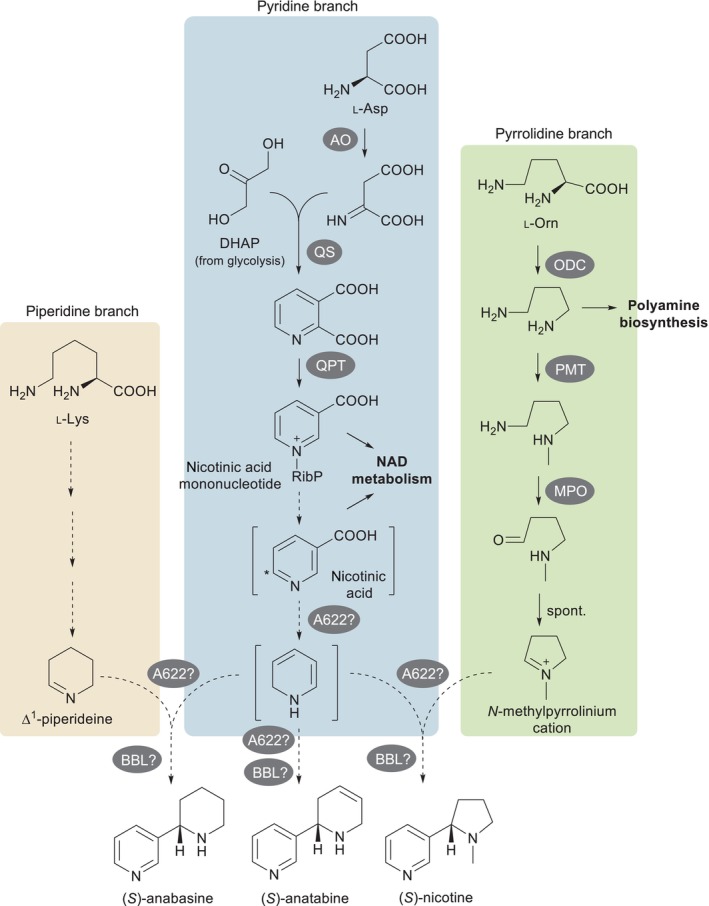
Schematic diagram of pyridine alkaloid biosynthesis in *Nicotiana* spp. The three colored boxes highlight the different biosynthetic branches involved. Full arrows represent single characterized steps, and dotted arrows represent one or more putative steps. The intermediates shown in brackets are putative. The asterisk in nicotinic acid indicates position C6, whose hydrogen is lost during biosynthesis (Dawson *et al*., 1960). Enzymes are represented by dark gray ovals. Question marks indicate uncertainty about the precise reactions catalyzed by the mentioned enzymes. A622, PIP‐family reductase; AO, aspartate oxidase; BBL, berberine bridge enzyme‐like protein; l‐Lys, l‐lysine; l‐Orn, l‐ornithine; MPO, methylputrescine oxidase; NAD, nicotinamide adenine dinucleotide; ODC, ornithine decarboxylase; PMT, putrescine methyltransferase; QPT, quinolinate phosphorybosyltransferase; QS, quinolinate synthase; RibP, ribose phosphate residue.

Nicotine biosynthesis occurs primarily in roots from where it is transported to aerial tissues via the xylem (Zenkner *et al*., 2019). On top of a basal level of production, nicotine biosynthesis can be induced by herbivore attack or by methyl jasmonate (MeJa) treatment (Steppuhn *et al*., 2004). The nicotine molecule is composed of two nitrogen‐containing rings coupled directly to each other: a pyridine ring likely derived from nicotinic acid and a pyrrolidine ring evolutionarily derived from polyamine biosynthesis (Xu *et al*., 2017; Fig. 1). The pyridine branch is well characterized up to nicotinic acid mononucleotide, from which nicotinic acid is thought to derive (Shoji & Hashimoto, 2011). The pyrrolidine branch is fully characterized and gives rise to the *N‐*methylpyrrolidinium cation, which is proposed to be coupled directly to a reduced and decarboxylated form of nicotinic acid (Shoji & Hashimoto, 2011). Little is known about the coupling step itself; however, precursor feeding experiments have established that the carbon atom bearing the substitution in nicotinic acid becomes the equivalent carbon in nicotine (Yang *et al*., 1965; Scott & Glynn, 1967; Leete, 1977).

Finally, the coupled intermediate likely undergoes oxidation to give the final nicotine molecule (Fig. 1). The details of these last steps of nicotine biosynthesis remain undefined; however, one important clue is that the hydrogen at position C6 of the pyridine ring in nicotinic acid is lost during biosynthesis (Dawson *et al*., 1960; Leete & Liu, 1973; Leete, 1978; Fig. 1). The related pyridine alkaloids anatabine and anabasine are also composed of two coupled nitrogen rings each; however, the composition of the nonpyridine rings varies. In the case of anatabine, both rings are derived from the pyridine branch, while in the case of anabasine, one ring comes from the pyridine branch and the second one comes from a piperidine branch originating from the amino acid l‐lysine (Fig. 1). All three pyridine alkaloids possess a stereocenter at position C2 of the nonpyridine ring. In *Nicotiana tabacum*, the *S* forms predominate (Armstrong *et al*., 1998, 1999), most notably for nicotine, where the enantiomeric fraction reaches *c*. 99.8% (Armstrong *et al*., 1999). In animals, the *S* enantiomer of nicotine is more toxic and displays greater pharmacological effects (Yildiz, 2004). The biosynthetic step that determines the *S* configuration remains to be determined, but it is conceivable that the coupling step is under stereoselective enzymatic control, thus mainly producing an *S* intermediate whose stereochemistry is retained during the final oxidation step. In *N. tabacum*, (*S*)‐nicotine accounts for 96% of the nicotine produced in the roots, and preferential demethylation of its enantiomer to give nornicotine accounts for an increased enantiomeric fraction of up to 99.8% (Cai *et al*., 2013). *Nicotiana benthamiana* plants accumulate lower amounts of nornicotine compared with *N. tabacum* (Sisson & Severson, 1990); thus, it is unclear whether this fine‐tuning mechanism operates in *N. benthamiana*.

Two enigmatic enzymes have been implicated in the late biosynthetic steps of all pyridine alkaloids. The first one is a reductase of the phosphatidylinositol phosphate (PIP) family called A622. Conditional suppression of two *A622* copies in *N. tabacum* hairy roots via RNAi resulted in cell growth inhibition, lower levels of pyridine alkaloids, and accumulation of a nicotinic acid *N‐*glucoside as well as the *N*‐methylpyrrolidinium cation (Kajikawa *et al*., 2009). This suggests that A622 catalyzes the putative reduction and decarboxylation of nicotinic acid and/or the coupling step (Kajikawa *et al*., 2009). However, there are no reports showing the catalytic properties of A622. The second implicated enzyme is a berberine bridge enzyme‐like protein (BBL). Simultaneous suppression of several *BBL* copies in *N. tabacum* plants and hairy roots via RNAi led to a reduction in pyridine alkaloid levels and accumulation of dihydrometanicotine (DMN; Kajikawa *et al*., 2011; Lewis *et al*., 2015). DMN itself does not appear to be a substrate of BBLs, as recombinant proteins expressed in *Pichia pastoris* exhibited no activity toward it (Kajikawa *et al*., 2011). However, this lack of activity could have resulted from incorrect post‐translational processing (Kajikawa *et al*., 2011). Further evidence for BBL involvement has come from the simultaneous inactivation of two or three *N. tabacum BBL* copies via chemical mutagenesis, which reduced the nicotine content up to 13‐fold (Lewis *et al*., 2015). Later, CRISPR/Cas9 technology was used to introduce mutations into all six *BBL* copies found in the *N. tabacum* genome, producing lines reported to be nicotine free (Schachtsiek & Stehle, 2019). By contrast, a follow‐up study on the chemically mutagenized double and triple *BBL* mutants showed that further inactivation of the remaining *BBL* genes by CRISPR/Cas9 technology did not result in any further nicotine reductions (Lewis *et al*., 2020).

While the involvement of A622 and BBL in nicotine biosynthesis is undisputed, the exact reactions that they catalyze are yet to be determined. In this study, we report an improved assembly of the *N. benthamiana* genome and identify five *BBL* genes. We use CRISPR/Cas9 technology to generate low‐nicotine lines with loss‐of‐function mutations in individual and multiple combinations of *BBL* genes. Surprisingly, analysis of lines with no functional BBL revealed that the residual nicotine was fully racemic. Furthermore, precursor feeding studies indicated that the mutants did not undergo the specific loss of C6 hydrogen as observed in the control lines. Our work provides an improved low‐nicotine *N. benthamiana* chassis for bioproduction and sheds light into the precise catalytic role of BBL enzymes in nicotine biosynthesis.

## Materials and Methods

### Plant growth

For DNA extraction, gene‐expression analysis, and seed production, *Nicotiana benthamiana* Domin plants were grown in a peat‐based potting mix (90% peat, 10% grit, supplemented with 4 kg m^−3^ dolomitic limestone, 0.75 kg m^−3^ powdered compound fertilizer, and 1.5 kg m^−3^ slow‐release fertilizer) in a controlled environment room with 16 h : 8 h, light : dark at 22°C, 80% humidity, and *c*. 200 μmol m^−2^ s^−1^ light intensity.

### Genome assembly

Genomic DNA was purified and sequenced using Illumina (San Diego, CA, USA), Chromium linked‐read, and Oxford Nanopore Technologies (Oxford, UK), and the genome was assembled as described in Supporting Information Methods S1.

### Expression analysis

For expression analysis, wild‐type plants were treated with a foliar spray of 2.5 mM MeJa in dimethyl sulfoxide (DMSO) or DMSO (control plants) 7 d before sampling. Total RNA was isolated from 100 mg of fresh root tissue sampled from three individual plants (biological replicates) using the Spectrum Plant RNA Purification Kit (Merck, Darmstadt, Germany). RNA was treated with RNase‐free DNase Set (Qiagen) and cDNA was synthesized from 300 ng of total RNA using the M‐MLV Reverse Transcriptase (Thermo Fisher Scientific, Waltham, MA, USA). SYBR® Green JumpStart™ Taq ReadyMix™ (Merck) was employed for reporting successful amplification using a QuantStudio 6 Pro real‐time PCR system (Applied Biosystems, Waltham, MA, USA) with specific primer pairs for each target *NbBBL* gene. Primer sequences used for expression analysis are provided in Table S1. *NbEF1a* was amplified as an internal reference (Liu *et al*., 2012) for relative gene expression. Four technical replicates were performed for each sample and primer pair.

### Cas9‐mediated targeted mutagenesis by *Agrobacterium*‐mediated transformation

A binary vector for Cas9‐mediated targeted mutagenesis via *A. tumefaciens*‐mediated transgenesis was assembled using the plant modular cloning toolkit (Engler *et al*., 2014) as previously described (Dudley *et al*., 2022b). Two single guide RNA (sgRNA) expression cassettes were assembled by amplifying the sgRNA scaffold with an extended stem (B. Chen *et al*., 2013) from plasmid pEPOR1CB0022 (#117537; Addgene, Watertown, MA, USA) and integrating the spacer sequence for each target as a 5′ extension of the forward primer. Primer sequences used to amplify sgRNAs are provided in Table S2. The resulting amplicons were assembled in Level 1 acceptors with an AtU626 promoter (pICSL90002, AtU6‐26, #68261; Addgene). The final construct was assembled by combining the two Level 1 sgRNA cassettes with cassettes for resistance to kanamycin pICSL11024 (#51144; Addgene) and constitutive expression of SpCas9 (pEPQD1CB0001, #185625; Addgene). All four Level 1 cassettes were assembled in a one‐step reaction into the Level 2 acceptor plasmid (pAGM4723, #48015; Addgene) as previously described (Dudley *et al*., 2022b). The efficacy of the resulting binary construct was tested by transient infiltration as described previously (Dudley *et al*., 2022b). The resulting constructs were transformed into *A. tumefaciens* strain AGL1 and used for transformation of *N. benthamiana* as described previously (Dudley *et al*., 2022a).

### Cas9‐mediated targeted mutagenesis using a TRV2 viral vector

Spacer sequences for selected targets were incorporated into pEPQD0KN0750 (#185627; Addgene) by Golden Gate assembly into AarI sites as described previously (Dudley *et al*., 2022a). Primer sequences are provided in Table S3. Constructs were transformed into *A. tumefaciens* GV3101 and infiltrated into transgenic *N. benthamiana* plants constitutively expressing Cas9 (Cas9 Benthe 193.22 T5 Homozygous; gratefully received from Dan Voytas) together with a strain containing pTRV1 (#148968; Addgene) as described previously (Ellison *et al*., 2020; Dudley *et al*., 2022a). Plants were allowed to grow for 13 wk before samples of leaf tissue were taken from two different stems.

### Genotyping of gene‐edited lines

Genomic DNA was isolated from the leaves of T_0_ plants generated by *Agrobacterium*‐mediated transformation or from leaves sampled from E_0_ plants infiltrated with TRV viral vectors as described previously (Dudley *et al*., 2022a). Target loci were amplified using a proof‐reading polymerase (Q5® High‐Fidelity DNA Polymerase; New England Biolabs, Ipswich, MA, USA; Phusion™ High‐Fidelity DNA Polymerase; Thermo Fisher Scientific) and primers flanking the target sites. Primer sequences used to genotype edited lines are provided in Table S4. Amplicons were sequenced by Sanger sequencing (Eurofins, Luxembourg City, Luxembourg). Seeds were harvested from primary transgenic plants (T_0_) in which mutations were detected at one or more target loci. Seeds from plants infiltrated with TRV viral vectors (E_0_ plants) were harvested from seed pods on stems in which mutations had been detected. The resulting T_1_ and E_1_ plants were grown, and the genotype of each target loci was confirmed. Amplicons with double peaks suggesting biallelic mutation were analyzed using the Inference of CRISPR Edits (ICE) tool (Synthego, https://ice.synthego.com/) and confirmed by Sanger sequencing of plasmid‐cloned fragments. Stable, nonchimeric lines with homozygous or biallelic mutations at one or more target loci were selected for further analysis and T_2_/E_2_ seed collection. The genotypes of all *BBL* genes were confirmed in the T_2_/E_2_ generation. For the biallelic lines in this generation, we observed the expected segregation of each locus, confirming the genotyping results from the previous generation.

### Leaf alkaloid analysis of gene‐edited lines

T_2_/E_2_ plants were grown in soil in a glasshouse under long‐day conditions (16 h light) and day : night temperatures of 20°C : 19°C. After 34 d, six 1 cm leaf disks per plant representing three different leaves (two leaf disks per leaf) were harvested. The leaf disks were snap‐frozen and stored at −70°C until further use (uninduced samples). Subsequently, alkaloid biosynthesis was induced by spraying leaves with 0.1% (v/v) MeJa (equivalent to 4.6 mM MeJa) MeJa solution containing 0.1% (v/v) Tween 20 (source of MeJA: Duchefa Biochemie, Haarlem, the Netherlands). Five days after induction, another six leaf disks were harvested from the same leaves as before, snap‐frozen, and stored at −70°C. Frozen leaf disks from uninduced and induced leaves were subjected to metabolite extraction, LC–MS analysis, and for three of the lines, (*R*)‐ and (*S*)‐nicotine analysis as described below and in Methods S2.

### Precursor feeding experiments

Seedlings were grown using a hydroponic system illustrated in Fig. S1. For each sample, 20 seeds were germinated and grown on cotton gauze (28 thread) glued onto ¾‐inch rubber O‐rings fitted onto the wells of 12‐well plates. Seedlings were grown under long‐day conditions (16 h light) in tap water that was refilled when needed. Clear plate lids were placed on the plates (touching the O‐rings) for the first 5 d. Alkaloid biosynthesis was induced 17 d after placing the seeds on the cotton gauze. For the induction, two filter papers with 5 μl MeJa (Duchefa Biochemie) each were placed next to the plates, and one large cover (clear plastic) was placed over all plates, facilitating a closed environment. Two days later, the tap water was replaced with either 0.8 mM D4‐nicotinamide (D4 98%; Cambridge Isotope Laboratories, Tewksbury, MA, USA) or 0.8 mM unlabeled nicotinamide, both solutions in tap water. The respective nicotinamide solutions were refilled 2 d after the start of the feeding (continuous feeding). Fresh MeJa was added to the filter papers at the start of the feeding and also during the refill. Seedlings were harvested 5 d after the commencement of feeding. All seedlings from one sample were pooled and rinsed three times with 1 ml ultrapure water. Rinsed seedlings were snap‐frozen and stored at −70°C. Frozen samples were subjected to metabolite extraction and LC–MS analysis as described below and in Methods S2.

### Root alkaloid analysis of gene‐edited lines

To examine DMN formation in roots, seedlings were grown in a hydroponic system (Fig. S1) as described above, except that 6‐well plates with 1¼‐inch O‐rings were used instead, as well as 40 seeds per sample. Thirty‐five days after placing the seeds on the cotton gauze, alkaloid biosynthesis was induced using the MeJa filter paper method described above. Three days after the start of the induction, the wells were refilled with tap water and fresh MeJa was added to the filter papers. The seedlings were harvested 5 d after the start of the induction. For harvesting, seedlings were rinsed six times with 500 μl ultrapure water, and the roots were cut off, snap‐frozen, and stored at −70°C. Frozen samples were subjected to metabolite extraction and LC–MS analysis as described below and in Methods S2.

### Metabolite extraction

Frozen samples of leaf disks, whole seedlings, or seedling roots were lyophilized using a freeze dryer (CooleSafe™; Scanvac, Lillerød, Denmark). Freeze‐dried tissues were homogenized using chrome balls and a TissueLyzer (Qiagen). Metabolites were extracted with 60% (v/v) methanol containing 0.06% (v/v) formic acid and either 75 ppm caffeine (for leaf disks) or 25 ppm caffeine (for whole seedlings and seedling roots). The proportions of dry tissue (mg) to extractant (μl) varied depending on the tissue source: 4 mg 100 μl^−1^ for leaf disks, 4 mg 300 μl^−1^ for whole seedlings, and 4 mg 1000 μl^−1^ for seedling roots. After 1 h incubation at room temperature with constant shaking (1200 rpm), samples were spun down for 1 min at 16 200 ***g***. Supernatants were diluted in ultrapure water at a proportion of 1 : 15 in the case of leaf disks or 1 : 5 in the case of whole seedlings and seedling roots. Diluted samples were filtered through a 0.22 μm PVDF filter (MultiScreen_HTS_ GV Filter Plate, 0.22 μm, clear, nonsterile; Merck Millipore, Billerica, MA, USA) and stored at −70°C until further analysis. Methanolic extracts were analyzed via reversed‐phase LC–MS as described in Methods S2.

## Results

### An *N. benthamiana* genome assembled from Chromium linked‐reads and ONT‐generated long reads

The use of Chromium linked‐reads and ONT long reads resulted in a genome assembly of 2995.0 Mb, consisting of 50 663 scaffolds, with an N50 of 14.0 Mb and a L50 of 67. The assembly has high completeness, as evidenced by a Busco completeness of 97.6% and a k‐mer completeness of 97.82%. Additionally, the assembly quality value (QV) score of 37.88 indicates good per‐base accuracy. Comparison of our assembly with a recently published assembly generated using Pacific BioScience Highly Accurate Long Read Sequencing chemistry (PacBio HiFi) and HiC (Omni‐C reads; Kurotani *et al*., 2023), indicates that our assembly is of comparable completeness and quality (Table S5). However, the HiFi+HiC assembly exhibits higher contiguity, as evidenced by the larger N50 and the smaller number of contigs. The assembly is available for download and can be accessed at the following link: https://opendata.earlham.ac.uk/opendata/data/Patron_2023‐03‐01_Nicotiana_benthamiana_genome_assembly/

### *Nicotiana benthamiana* encodes five *BBL* genes

**Fig. 2:**
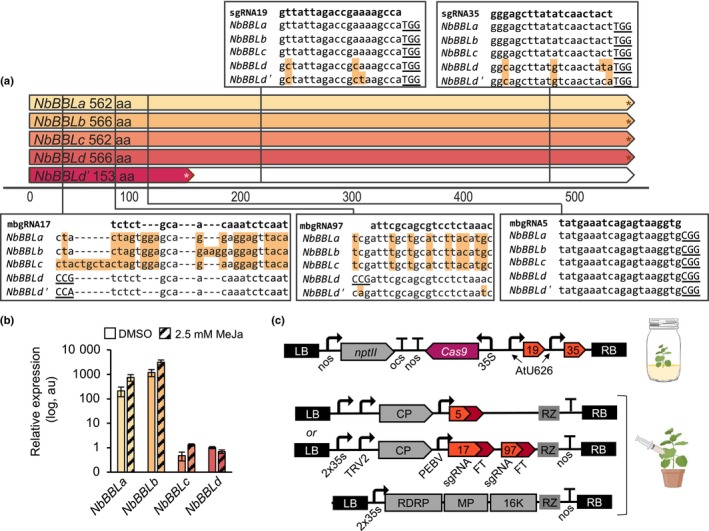
*Nicotiana benthamiana* berberine bridge enzyme‐like (NbBBL) enzymes. (a) Schematic of NbBBL enzymes indicating the locations and sequences of guide RNAs used for Cas9‐mediated targeted mutagenesis. Canonical NGG protospacer adjacent motifs are underlined; *, stop codon. Background shading of bases indicates a mismatch to the guide RNA sequence. (b) Expression of *NbBBL* genes relative to *NbEF1a* in roots of plants following treatment with 2.5 mM methyl jasmonate (MeJa). Expression was determined by reverse transcription‐quantitative PCR (RT‐qPCR). Error bars indicate the SE of the mean of three biological replicates (four technical replicates per sample). (c) Schematics of plasmid constructs used for Cas9‐mediated targeted mutagenesis by integration of T‐DNA (above) or transient expression of a mobile guide RNA (mbgRNA). 16k, 16k gene; 35s, cauliflower mosaic virus 35s promoter; CP, coat protein; FT, flowering locus T mobile signal; LB, left border; MP, movement protein; nos, nopaline synthase promoter or terminator; ocs, octopine synthase terminator; PEBV, pea early browning virus promoter; RB, right border; RDRP, RNA‐dependent RNA polymerase; RZ, self‐cleaving ribozyme. This figure contains graphics from Biorender (biorender.com).

Using the *NtBBL* genes as queries, we identified five candidate *BBL* genes in the *N. benthamiana* genome, four of which (*NbBBLa‐d*) encoded open reading frames that translate to proteins of similar length to the BBL proteins encoded in the *N. tabacum* genome. The fifth gene, *NbBBLd′*, contained a premature stop codon (Figs 2a, S2). Phylogenetic analysis indicates that *NbBBLa‐d* may be orthologues of the equivalently named genes in *N. tabacum*, but there is no obvious orthologue for *NtBBLe* in the *N. benthamiana* genome (Fig. S2). Identical genes were found in the PacBio HiFi assembly. The two older genomes based on short reads have identical sequences for *NbBBLc*, *NbBBLd*, and *NbBBLd*′, but there were discrepancies between the two assemblies with respect to *NbBBLa* and *NbBBLb*, potentially resulting from misassembly.

We analyzed the expression levels of *NbBBLa–d* in root tissues using reverse transcription‐quantitative (real‐time) polymerase chain reaction (RT‐qPCR). *NbBBLa* and *NbBBLb* were expressed at a higher level than *NbBBLc* and *NbBBLd* (Fig. 2b) and the expression of all genes except *NbBBLd* increased following exposure of plants to MeJa (Fig. 2b).

### Production of *N. benthamiana* lines with Cas9‐mediated targeted mutations in *NbBBL* genes

**Fig. 3:**
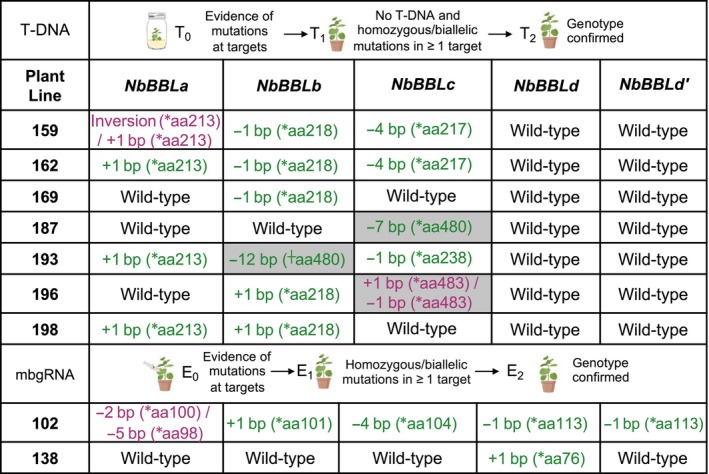
Workflow for the selection of gene‐edited lines, and genotypes of all *Nicotiana benthamiana berberine bridge enzyme‐like* (*NbBBL*) genes in each line. The size of insertions (+) and deletions (−) in base pairs (bp) are provided together with the last correct amino acid (*aa). +, 5 residues of *NbBBLb* AYINY (Alanine, Tyrosine, Isoleucine, Asparagine, Tyrosine) are replaced with D (Aspartic Acid). Green text, homozygous mutation; purple text, biallelic mutation; gray shading, due to the size, type or location, the mutation may not result in loss of activity. Details of all mutations can be found in Supporting Information Tables S6–S10. This figure contains graphics from Biorender (biorender.com).

We first constructed a binary vector expressing two single guide RNAs (sgRNA19 and sgRNA35) for the stable transformation of wild‐type *N. benthamiana* plants to introduce Cas9‐mediated targeted mutations in *NbBBLa‐c* (Fig. 2a,c). The genotypes of transgenic (T_0_) plants were determined by PCR amplification and sequencing of target loci. Lines in which the genotype was unclear or indicated genetic chimerism were discarded, and T_1_ seed was collected from lines with homozygous, heterozygous or biallelic mutations in one or more *NbBBL* genes. The genotype at each locus as well as the presence or absence of the T‐DNA was analyzed in T_1_ plants. T_2_ seeds were collected from plants in which the T‐DNA had segregated away and that contained homozygous or biallelic mutations at one or more *NbBBL* genes. Genotypes were confirmed in T_2_ plants and are provided in Fig. 3 and Tables S6–S10. We also constructed two TMV‐based viral vectors encoding mobile guide RNAs (mbgRNA) as previously described (Ellison *et al*., 2020). The first vector contained a single mbgRNA, mbgRNA5, targeting a single locus present in all *NbBBL* genes (Fig. 2a,c). A second vector contained two mbgRNAs, mbgRNA17 and mbgRNA97, targeting sequences unique to *NbBBLd/d′* (Fig. 2a,c). These vectors were infiltrated into the leaves of transgenic plants constitutively expressing Cas9, and progeny seeds were collected from pods that formed on stems in which mutations were detected at the target sites (see the Materials and Methods section). The genotypes of E_1_ plants were analyzed, and individuals with homozygous or biallelic mutations at loci of interest were selected (Fig. 3; Tables S6–S10). Overall, all guide RNAs except for mbgRNA17 resulted in mutations at the target locus. From these two methods, we were able to obtain lines with mutations in NbBBLb, NbBBLc, or NbBBLd alone, as well as lines with mutations in combinations of two, three, or five genes (Fig. 3; Tables S6–S10).

### Inactivation of *NbBBL* genes leads to low pyridine alkaloid levels

**Fig. 4:**
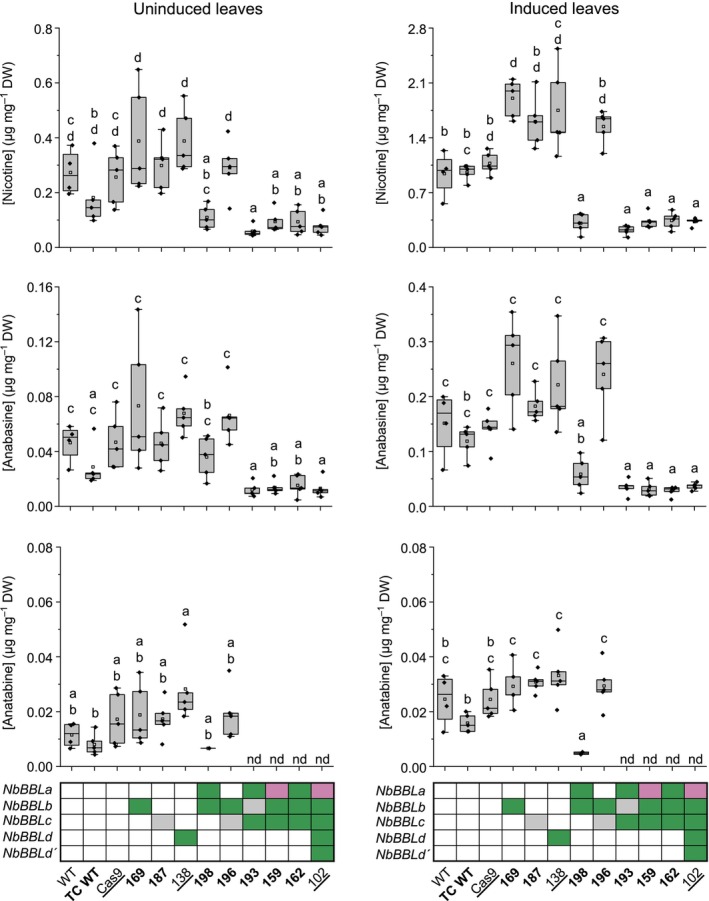
Pyridine alkaloid content in leaves of *Nicotiana benthamiana* berberine bridge enzyme‐like (*BBL*) mutant lines in comparison with control lines, as analyzed by LC–MS. Line names and their respective *NbBBL* genotypes are shown at the bottom, with green shading representing a homozygous knockout, purple shading indicating a biallelic knockout, and gray shading indicating uncertainty with respect to loss of function (due to size, type or location of the mutation; see details of all mutations in Fig. 3; Supporting Information Tables S6–S10). Three control lines were analyzed: WT, wildtype (control for all lines); TC WT, tissue culture control (additional control for lines 159–198; bold); and Cas9, transgenic line constitutively expressing Cas9 (additional control for lines 102 and 138; underlined). Graphs to the left represent the levels of nicotine, anabasine, and anatabine in leaves of glasshouse‐grown plants (nd, not determined). Graphs to the right represent the analogous measurements 5 d after induction with 0.1% (v/v) MeJa (equivalent to 4.6 mM MeJa). For each line, four to five biological replicates were measured (black diamonds). Compact letter display is used to visualize significant differences determined using ANOVA and *post hoc* Tukey tests on log‐transformed data. Adjusted *P*‐values for all pairs can be found in Table S11.

We analyzed the levels of pyridine alkaloids in leaves of the different *NbBBL* mutant lines (T_2_/E_2_ generation) using LC–MS. We quantified nicotine, anabasine, and anatabine, whereas nornicotine was not detectable (at least under our working dilutions). We included three different control lines: a wild‐type line (WT; control for all lines), a tissue culture control (TC WT; a wild‐type line recently regenerated in tissue culture, used as additional control for lines 159–198), and a transgenic Cas9 line (Cas9; the parental line constitutively expressing Cas9, used as additional control for lines 102 and 138; Fig. 4; Table S11). In general, the levels of nicotine were always the highest, followed by anabasine and then anatabine. Single *NbBBLb*, *NbBBLc*, and *NbBBLd* mutants did not differ from control lines, and neither did the double *NbBBLb/c* mutant. Gratifyingly, the double *NbBBLa/b* mutant and all higher order mutants displayed lower nicotine levels under MeJa induction compared with the respective control lines. Triple *NbBBLa/b/c* and quintuple *NbBBLa/b/c/d/d′* mutants did not appear to vary in nicotine content compared with the double *NbBBLa/b* mutant. One of the triple mutant lines (line 193) and the quintuple mutant displayed significantly lower nicotine content under both induced and uninduced conditions compared with the control lines (*c*. three‐ to fourfold reduction). The overall picture for anabasine and anatabine was similar. However, the anatabine levels in the triple and quintuple mutants were below our lowest calibration point and thus were not quantified (Fig. 4).

### Inactivation of *NbBBL* genes leads to a racemic nicotine mixture

**Fig. 5:**
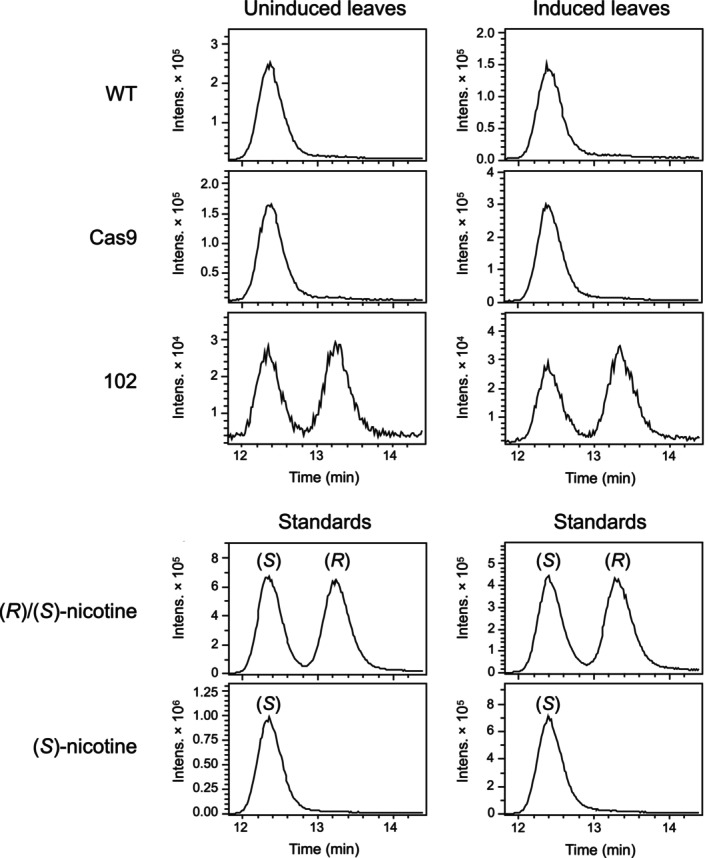
Analysis of (*S*)‐ and (*R*)‐nicotine in leaves of the quintuple *Nicotiana benthamiana* berberine bridge enzyme‐like (*NbBBL*) mutant (line 102) in comparison with control lines (WT and Cas9). Traces correspond to extracted ion chromatograms (nicotine, [M + H]^+^) resulting from chiral LC–MS analyses (Lux® 3 μm AMP column). Traces on the left are from uninduced plants, while traces on the right are from the same plants 5 d after induction with MeJa. Higher injection volumes were used for all uninduced samples (10 μl compared with 2 μl) to obtain comparable peak sizes. A total of four to five biological replicates were analyzed. While only one replicate is shown in this figure, all data are provided in Supporting Information Fig. S3. The two bottom rows show the results of running a racemic nicotine standard and a (*S*)‐nicotine standard along with the uninduced and induced samples.

The analysis of pyridine alkaloids described above did not discriminate between *S* and *R* enantiomers. Thus, we switched to chiral LC–MS analysis to determine whether inactivation of *NbBBL* genes had any effect on enantiomeric purity. We used a method able to separate (*S*)‐ and (*R*)‐nicotine with near baseline resolution, and we focused on two control lines (wildtype and Cas9 transformant) vs the quintuple *NbBBLa/b/c/d/d′* mutant (line 102). While the control lines accumulated (*S*)‐nicotine almost exclusively, the residual nicotine in the quintuple mutants was racemic (Figs 5, S3).

### Inactivation of *NbBBL* genes prevents hydrogen loss from position C6

As mentioned in the introduction, feeding experiments with isotopically labeled nicotinic acid have shown the loss of hydrogen at position C6 during its incorporation into nicotine in *N. tabacum* (Dawson *et al*., 1960). Subsequent publications have supported this finding for nicotine biosynthesis (Leete & Liu, 1973) and extended it to anatabine and anabasine biosynthesis (Leete, 1978). Furthermore, nicotinamide has been found to incorporate into nicotine to a similar extent as nicotinic acid (Dawson *et al*., 1960).

**Fig. 6:**
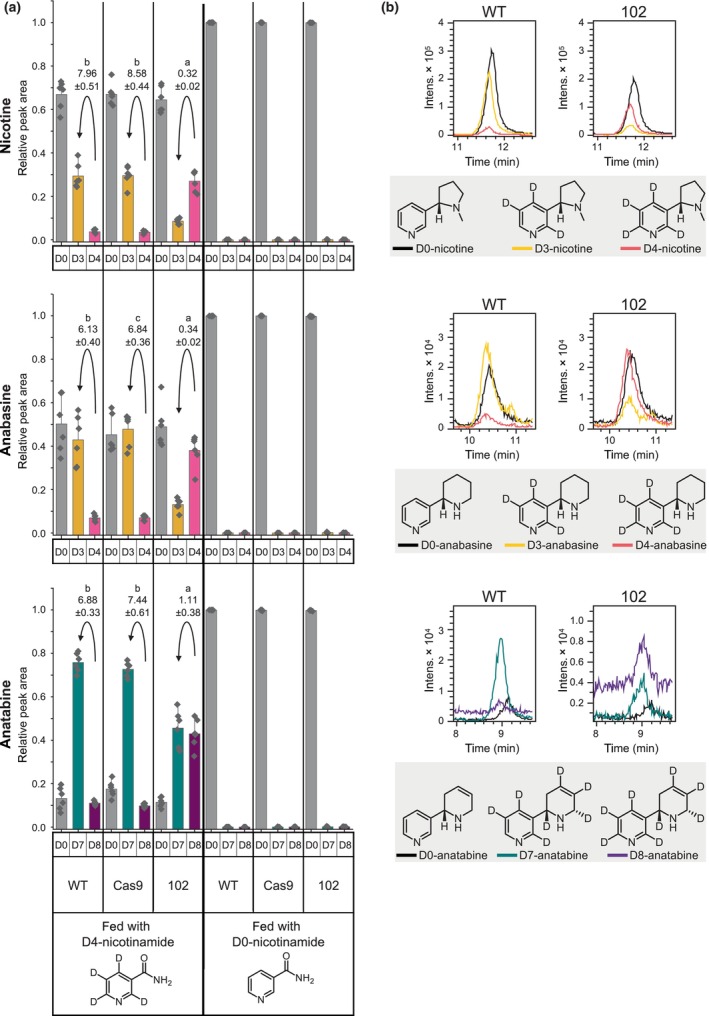
Incorporation of deuterated (D4)‐nicotinamide into pyridine alkaloids in the quintuple *Nicotiana benthamiana* berberine bridge enzyme‐like (*NbBBL*) mutant (line 102) in comparison with control lines (WT and Cas9). Seedlings were grown under hydroponic conditions, induced with MeJa, and fed continuously for 5 d with either labeled (D4) or unlabeled (D0, control) nicotinamide via the roots. (a) Relative levels of differently labeled nicotine, anabasine, and anatabine in whole seedlings, as observed using LC–MS. For nicotine and anabasine, D0, D3, and D4 versions were observed and quantified. For anatabine, D0, D7, and D8 versions were observed and quantified. For each version, relative peak areas were obtained by dividing by the sum of peak areas of all labeled versions. Six biological replicates were analyzed (black diamonds). Bars represent mean values, and error bars represent the outlier range in plus direction with a coefficient of 1.5. Numbers above the arrows correspond to the fold difference between D3 and D4 versions (for nicotine and anabasine) or between the D7 and D8 versions (for anatabine), ± SD. Compact letter display is used to visualize significant differences determined using ANOVA and *post hoc* Tukey tests on log‐transformed data. Adjusted *P*‐values for all pairs can be found in Supporting Information Table S12. (b) Representative traces (extracted ion chromatograms, [M + H]^+^) of the differently labeled pyridine alkaloids in quintuple *NbBBL* mutants (line 102) compared with WT plants. The colors of the depicted traces correspond to the colors of the corresponding bars in panel a. Chemical structures of each of the analyzed compounds are shown inside the gray boxes under each pair of graphs. The position of the lost deuterium atom was inferred from the comprehensive feeding studies carried out previously (Dawson *et al*., 1960; Leete & Liu, 1973; Leete, 1978).

We examined the incorporation of labeled D4‐nicotinamide into nicotine, anabasine, and anatabine in the two control lines (wildtype and Cas9 transformant) vs the quintuple *NbBBLa/b/c/d/d′* mutant, all lines under MeJa induction. We observed that the main labeled form of nicotine and anabasine in the fed control lines contained three deuterium atoms (D3 versions), demonstrating the loss of one deuterium atom during incorporation (Fig. 6; Table S12). By contrast, the quintuple mutant contained predominantly D4‐nicotine and D4‐anabasine instead of the corresponding D3 versions. For anatabine, in which both rings derive from the pyridine branch, we detected the D7 version predominantly, whereas D7 and D8 versions accumulated equally in the quintuple mutant (Fig. 6). Interestingly, while only *c*. 30% of nicotine was found labeled in these experiments, the percentage increased to *c*. 50% in the case of anabasine, and *c*. 80% in the case of anatabine (Fig. 6; Table S12).

### Inactivation of *NbBBL* genes leads to accumulation of DMN

**Fig. 7:**
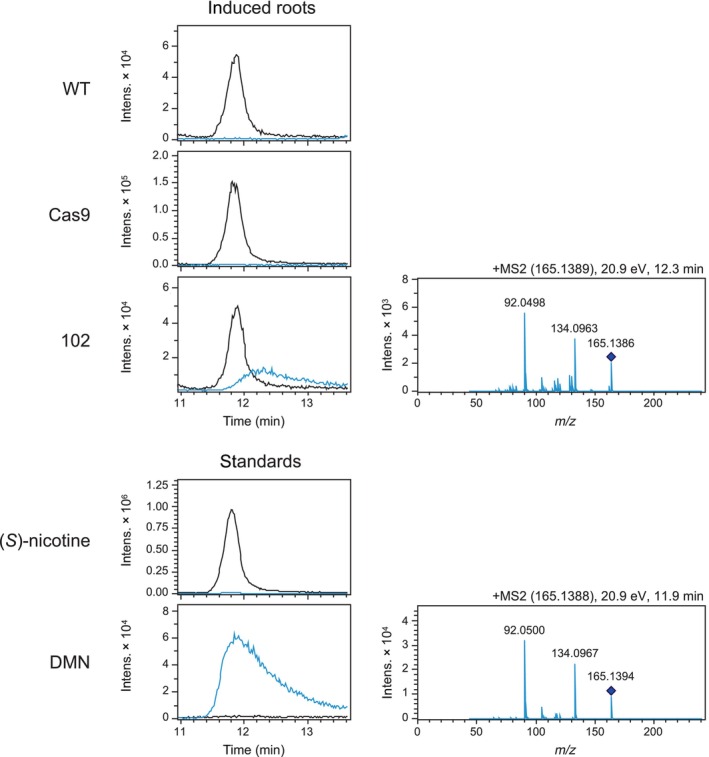
Dihydrometanicotine (DMN) accumulation in roots of the quintuple *NbBBL* mutant (line 102) as analyzed by LC–MS. Seedlings were grown under hydroponic conditions, and roots were harvested 5 d after induction with MeJa. Traces are extracted ion chromatograms ([M + H]^+^) corresponding to nicotine (black) or DMN (blue). Results from mutant line 102 are compared with results from two control lines (WT and Cas9). A total of five to six biological replicates per line were analyzed. While only one replicate is shown in this figure, all data is provided in Supporting Information Fig. S4. Also shown are two traces corresponding to a (*S*)‐nicotine standard and a DMN standard. In addition, MS2 spectra for DMN ([M + H]^+^) from line 102 and from the DMN standard are shown to the right. The labels at the top of the MS spectra indicate the mass of the fragmented ion (parenthesis), the collision energy, and the retention time.

Apart from displaying low‐nicotine levels, *N. tabacum* RNAi lines downregulated in *BBL* genes accumulate DMN in biosynthetic tissues (Kajikawa *et al*., 2011; Lewis *et al*., 2015). To examine whether a similar accumulation occurred in our gene‐edited *N. benthamiana* plants, we harvested MeJa‐induced roots from hydroponically grown quintuple mutants (line 102) and analyzed them via LC–MS. DMN was unequivocally detected in the mutant roots, while the compound was undetectable in the control lines (WT and Cas9; Figs 7, S4).

## Discussion

In this study, we report the production and analysis of low‐nicotine *N. benthamiana* lines using CRISPR/Cas9 to inactivate combinations of *BBL* genes, which are known to be involved in pyridine alkaloid biosynthesis. Our choice of *BBL* genes mirrors the choice taken by Lewis *et al*. (2015) when aiming at low‐nicotine *N. tabacum* lines. Briefly, aiming at genes in the pyrrolidine branch (e.g. *PMT* or *MPO*) is likely to result in lower nicotine levels at the expense of increased anatabine levels (Chintapakorn & Hamill, 2003; Wang *et al*., 2009). Conversely, aiming at genes involved only in the pyridine branch (e.g. *QPT*) may interfere with NAD metabolism (Lewis *et al*., 2015). *A622* and *BBL* genes would seem to be suitable targets based on their involvement in the late pathways toward the three pyridine alkaloids. However, Kajikawa *et al*. (2009) reported a failure to obtain *N. tabacum* lines constitutively silenced in *A622*, possibly due to the accumulation of nicotinic acid. It should also be noted that two transcription factors represented by the *nic1* and *nic2* loci are known to control nicotine biosynthesis in *N. tabacum* (Shoji *et al*., 2010; Shoji & Hashimoto, 2012; Qin *et al*., 2021). These loci are not commonly used in tobacco breeding due to lower yields (Legg *et al*., 1970; Chaplin & Weeks, 1976) likely caused by overaccumulation of polyamines (Nölke *et al*., 2018). Thus, *BBL* genes seemed to be the best targets for our purposes in *N. benthamiana*.

Our first task was the identification of all *BBL* gene copies in *N. benthamiana*. At the start of our work, there were two published *N. benthamiana* genome drafts, both of which had identified several examples of homeologous genes consistent with an allotetraploid origin (Bombarely *et al*., 2012; Naim *et al*., 2012). However, these genome drafts were constructed from reads generated using Illumina‐chemistry and, due to the complex nature of the genome, the assemblies consisted of > 140 000 scaffolds. To obtain an improved draft, we generated a new *N. benthamiana* genome assembly using Chromium™ linked‐read sequencing (10× Genomics) as well as Nanopore sequencing (Oxford Nanopore). The completeness of the assembly is comparable to a recently reported draft assembled from Pacific BioScience Highly Accurate Long Read Sequencing reads (PacBio HiFi; Kurotani *et al*., 2023); however, the HiFi+HiC assembly has higher contiguity (Table S5). This comparison provides insights into the limitations and advantages of different sequencing technologies, which will inform future efforts in genome assembly and analysis and highlights the benefits of chromosome‐spanning connectivity information. The availability of multiple long‐read datasets provides the opportunity for further improvements by the community.

Out of the five *NbBBL* genes identified in the *N. benthamiana* genome, four were full‐length (*NbBBLa*, *b*, *c*, and *d*), three were MeJa‐responsive (*NbBBLa*, *b*, and *c*), and two were expressed at high levels (*NbBBLa* and *b*; Fig. 2a,b). We carried out gene editing of *NbBBL* genes using two different approaches. The first was a traditional approach targeting *NbBBLa*, *b*, and *c*, by integrating a T‐DNA encoding a pair of sgRNAs to maximize the chances of inducing loss‐of‐function mutations (sgRNA19 and 35; Fig. 2a,c). We isolated a number of transgene‐free lines with mutations in at least one of the two sgRNA sites, including a double *NbBBLa/b* knockout (line 198) and at least two triple *NbBBLa/b/c* knockouts (lines 159 and 162; Fig. 3). For the second gene‐editing approach, we used virus‐delivered mobile guide RNAs (mbgRNAs; Ellison *et al*., 2020) which, in one embodiment, targeted all *BBL* genes (mbgRNA5; Fig. 2a,c). With this strategy, we succeeded in obtaining a quintuple knockout (line 102).

Metabolite analysis in leaves of the mutant lines revealed that simultaneous inactivation of *NbBBLa* and *NbBBLb* is sufficient to lower the levels of pyridine alkaloids, at least under induced conditions (Fig. 4). Further inactivation of *NbBBLc*, *NbBBLd*, and *NbBBLd′* did not result in significantly lower levels of pyridine alkaloids (Fig. 4). Together with the above‐mentioned gene expression results (Fig. 2b), this indicates that *NbBBLa* and *NbBBLb* are the main *NbBBL* genes involved in pyridine alkaloid biosynthesis in *N. benthamiana*. A major role of *BBLa* and *BBLb* was also observed in *N. tabacum* (Lewis *et al*., 2015, 2020; Kajikawa *et al*., 2017). Indeed, Kajikawa *et al*. (2017) reported that *NtBBLa* and *NtBBLb* are by far the highest expressed *NtBBLs* in cultivar TN90. Consistent with this, Lewis *et al*. (2015) observed that double *NtBBLa/b* and triple *NtBBLa/b/c* knockouts (EMS‐induced) in cultivar DH98‐325‐6 displayed comparably reduced levels of nicotine, nornicotine, and anatabine. A follow‐up publication by Lewis *et al*. (2020) supported these findings by transferring the mutant alleles to different backgrounds (cultivars K326, TN 90, and TN90 SRC). Similar to our results with the quintuple *N. benthamiana* knockout, Lewis *et al*. did not observe further pyridine alkaloid reductions by inactivating the remaining *NtBBL* genes (*d1*, *d2*, and *e*) on top of the triple *NtBBLa/b/c* mutant (TN90 SRC background).

In the light of both our study and the one by Lewis *et al*. (2020), it is puzzling that the multiple *NtBBL* knockout reported in a brief communication by Schachtsiek & Stehle (2019) did not accumulate any detectable nicotine. We set out to investigate the origin of the residual nicotine in our *NbBBL* knockouts. Chiral analysis revealed that, while control lines accumulated (*S*)‐nicotine almost exclusively, the residual nicotine in our quintuple *NbBBL* knockout was fully racemic (Fig. 5). Not only does this suggest that BBLs are fully responsible for the chirality of nicotine, it also suggests that the residual nicotine derives from a nonstereoselective, spontaneous reaction. In addition, we carried out precursor feeding studies with deuterated (D4) nicotinamide. In control plants, we observed the loss of one deuterium atom, leading to preferential accumulation of D3 compared with D4 pyridine alkaloid versions (Fig. 6). This preferential accumulation was lost in the quintuple *NbBBL* knockout (Fig. 6), strongly supporting the hypothesis of a spontaneous reaction. It is well known that, in biological systems, spontaneous reactions are sometimes made faster and/or more specific via the action of enzymes. In particular, some of the scaffold‐forming reactions in plant alkaloid biosynthesis are subject to enzymatic control despite their spontaneous nature, as discussed recently by Lichman (2021). One example is the coupling of tryptamine and secologanin in the biosynthesis of monoterpene indole alkaloids. In this case, the spontaneous reaction is slow and gives rise to two stereoisomers, whereas the enzymatic reaction is stereoselective, giving rise only to the *S* product (Stöckigt & Zenk, 1977). In a similar fashion, we postulate that BBLs not only accelerate an otherwise spontaneous reaction, but also ensure exclusive accumulation of the *S* stereoisomer of nicotine, which is the more biologically active one (Yildiz, 2004).

**Fig. 8:**
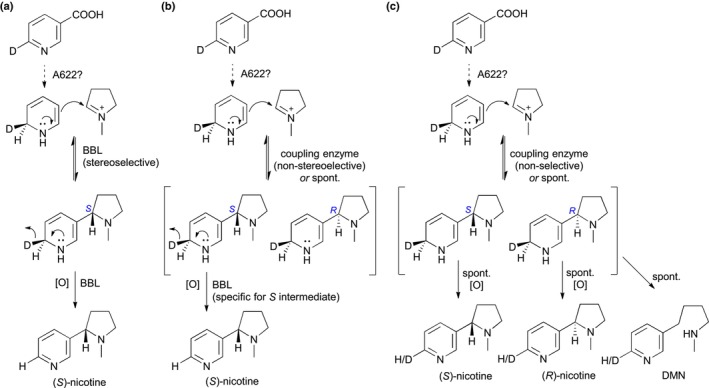
Mechanistic proposals for the BBL‐catalyzed reaction(s). The proposals are based on our experimental results with the quintuple *NbBBL* knockout as well as previous results by Dawson and Leete (Dawson *et al*., 1960; Leete & Liu, 1973; Leete, 1978). A deuterium atom is shown at the C6 position in nicotinic acid (top molecule) to facilitate visualization of the fate of a hydrogen isotope at this position during biosynthesis. The stereochemistry of the initial reduction was established by Leete (1978). In the BBL‐catalyzed oxidations, the deuteride acceptor is likely to be FAD (not shown). [O], oxidation; DMN, dihydrometanicotine; spont., spontaneous. (a) Scenario in which BBL enzymes catalyze both a stereoselective coupling and a subsequent oxidation. (b) Alternative scenario in which the coupling is not stereoselective and is either spontaneous or catalyzed by an unknown enzyme. In this scenario, BBL enzymes are specific for the *S* intermediate, thus giving rise only to (*S*)‐nicotine. (c) Proposal for the pyridine alkaloid pathway in the absence of functional BBL enzymes.

One outstanding question remains: what is the precise reaction catalyzed by BBL enzymes? As mentioned before, localized accumulation of DMN has been observed in *N. tabacum* roots upon down‐regulation or inactivation of *NtBBL* genes (Kajikawa *et al*., 2011; Lewis *et al*., 2015). We observed a similar accumulation of DMN in our quintuple *NbBBL* mutant, confirming that the catalytic role of BBLs is conserved between *N. tabacum* and *N. benthamiana*. Given that DMN does not appear to be a substrate and that it likely derives from an intermediate downstream of the coupling step, Kajikawa *et al*. (2011) entertained the possibility that BBLs catalyze the oxidation of an already coupled, unstable intermediate (Kajikawa *et al*., 2011). This proposal is fully consistent with our precursor feeding experiments, which showed a BBL‐dependent loss of hydrogen from the pyridine ring. It is also consistent with several known BBL‐catalyzed reactions involving hydride removal next to a nitrogen atom (Daniel *et al*., 2017). However, the proposal seems to be at odds with the observed BBL‐dependent creation of the *S* stereocenter in the pyrrolidine ring. The inconsistency stems from the fact that the stereocenter is likely established during the coupling. To bridge this gap, we propose a double function where BBLs catalyze the stereoselective coupling as well as the subsequent oxidation, as shown in Fig. 8(a). Yet, stereochemical control of the coupling reaction is not the only way BBL enzymes can be envisioned to direct nicotine biosynthesis toward the *S* form. In case of a non‐BBL‐catalyzed, nonstereoselective coupling leading to both *S* and *R* intermediates, BBL enzymes would still produce only (*S*)‐nicotine if they were specific for the *S* intermediate. We show this alternative proposal in Fig. 8(b). In this scenario, an *R* intermediate would be produced, but it would be in fast equilibrium with the noncoupled forms, thus eventually converting to (*S*)‐nicotine via decoupling.

Both of these proposals are consistent with our results, especially in light of the coupling reaction having an important degree of spontaneous character and the coupled intermediates being unstable. Upon inactivation of BBL enzymes as in our quintuple *NbBBL* mutant, the coupling would still occur (either spontaneously or catalyzed by an unknown enzyme), leading to production of both *S* and *R* intermediates. Apart from decoupling (reverse reaction), each coupled intermediate would have two possible fates: either oxidize spontaneously to the corresponding nicotine form, or undergo ring‐opening to give DMN (Fig. 8c). In all of these cases, the loss of a hydrogen from position C6 would be nonspecific, precluding the preferential accumulation of D3 forms in the case of feeding with D4‐nicotinamide. Moreover, if hydrogen loss during oxidation was rate limiting, D4 forms of the pyridine alkaloids would predominate due to the kinetic isotope effect, and this is indeed what we observed for nicotine and anabasine (Fig. 7).

If the reaction(s) catalyzed by BBLs can indeed occur spontaneously, no further reduction in pyridine alkaloids can be expected by targeting this pathway step. A promising approach for further reduction could be the stacking of mutations in the Nic1/Nic2 loci as described in *N. tabacum* by Lewis *et al*. (2020). Indeed, combining their *NtBBL*a/b/c alleles with the existing *nic2* locus resulted in a line with lower nicotine levels, and these were further reduced when *nic1* was stacked on top (*NtBBLa/b/c/nic1/nic2* line; Lewis *et al*., 2020). As mentioned before, the *nic1/nic2* loci are associated with reduced leaf quality in tobacco. This trait is associated with delayed senescence, which is problematic for the tobacco industry, but may be acceptable and even desirable in the case of *N. benthamiana*. However, the increased levels of polyamines observed in *N. tabacum* (Nölke *et al*., 2018) would be undesirable for high‐value compound production in *N. benthamiana*.

In summary, we present an improved *N. benthamiana* genome draft as well as low‐nicotine lines edited in *NbBBL* genes. Further analysis of edited lines provided insights into the nature of the reaction catalyzed by BBLs and uncovered their role in the stereoselectivity of nicotine biosynthesis.

## Competing interests

None declared.

## Author contributions

TY, QMD, FG‐F and NJP conceptualized the study. QMD and DH extracted genomic DNA and performed nanopore sequencing. MOF, GL and DS assembled the genome. QMD and MTO performed gene expression experiments. TY designed and assembled plasmid constructs for stable transformation. QMD designed, assembled, and delivered viral vectors for mutagenesis. MC and MAS performed all plant tissue culture with supervision from WAH. QMD and KV performed genotyping of edited plants. KV and DM performed the analysis of alkaloid content. KV conducted the precursor feeding experiments and investigated the stereochemistry of nicotine and the accumulation of DMN. FG‐F and NJP were responsible for supervision and funding acquisition. KV, QMD, MOF, FG‐F and NJP drafted the text and figures. All authors contributed to revising and editing the text. KV and QMD contributed equally to this work.

## Supporting information


**Fig. S1** Schematic and photographs of the hydroponic system.
**Fig. S2** Maximum likelihood tree and multiple sequence alignment of *Nicotiana tabacum* and *Nicotiana benthamiana BBL* genes.
**Fig. S3** Analysis of (*S*)‐ and (*R*)‐nicotine in leaves of the quintuple *NbBBL* mutant (line 102) in comparison with control lines (WT and Cas9).
**Fig. S4** Dihydrometanicotine DMN accumulation in roots of the quintuple *NbBBL* mutant (line 102) in comparison with two control lines (WT and Cas9), as analyzed by LC–MS.
**Methods S1** Genome sequencing and assembly.
**Methods S2** Liquid chromatography–mass spectrometry (LC*–*MS) analysis of methanolic extracts.
**Table S1** Primers used for expression analysis of *Nicotiana benthamiana berberine bridge‐like* (*NbBBL*) genes.
**Table S2** Primers used for amplification of sgRNA scaffolds.
**Table S3** Primers used for construction of the mobile single guide RNA plasmid vectors.
**Table S4** Primers used for genotyping *Nicotiana benthamiana* plants with Cas9‐induced mutations.
**Table S5** Comparison of *Nicotiana benthamiana* genome assemblies.
**Table S6** NbBBLa genotypes in *Nicotiana benthamiana* plants with Cas9‐mediated mutations.
**Table S7** NbBBLb genotypes in *Nicotiana benthamiana* plants with Cas9‐mediated mutations.
**Table S8** NbBBLc genotypes in *Nicotiana benthamiana* plants with Cas9‐mediated mutations.
**Table S9** NbBBLd genotypes in *Nicotiana benthamiana* plants with Cas9‐mediated mutations.
**Table S10** NbBBLd′ genotypes in *Nicotiana benthamiana* plants with Cas9‐mediated mutations.
**Table S11** Adjusted *P*‐values (single‐step method) of ANOVA and *post hoc* Tukey tests for Fig. 4.
**Table S12** Adjusted *P*‐values (single‐step method) of ANOVA and *post hoc* Tukey tests for Fig. 6.
Please note: Wiley is not responsible for the content or functionality of any Supporting Information supplied by the authors. Any queries (other than missing material) should be directed to the *New Phytologist* Central Office.

## Data Availability

The assembled genome is available at: https://opendata.earlham.ac.uk/opendata/data/Patron_2023‐03‐01_Nicotiana_benthamiana_genome_assembly. Reads are available under accession nos.: ERR3971933, ERR3971934, ERR3971506, ERR3971507, ERR3971508, ERR3971509, ERR3972081, ERR3972082, ERR3972083, ERR3972084, ERR3972085 and ERX10379414.
