# Supplementary material for: An improved Nicotiana benthamiana bioproduction chassis provides novel insights into nicotine biosynthesis
Source: New Phytol. 2023 Jul 24;240(1):302–17. doi: 10.1111/nph.19141 (PMC10952274; doi:10.1111/nph.19141)
Supplement: Supplementary file 1 — Fig. S1 Schematic and photographs of the hydroponic system. Fig. S2 Maximum likelihood tree and multiple sequence alignment of Nicotiana tabacum and Nicotiana benthamiana BBL genes. Fig. S3 Analysis of (S)‐ and (R)‐nicotine in leaves of the quintuple NbBBL mutant (line 102) in comparison with control lines (WT and Cas9). Fig. S4 Dihydrometanicotine DMN accumulation in roots of the quintuple NbBBL mutant (line 102) in comparison with two control lines (WT and Cas9), as analyzed by LC–MS. Methods S1 Genome sequencing and assembly. Methods S2 Liquid chromatography–mass spectrometry (LC–MS) analysis of methanolic extracts. Table S1 Primers used for expression analysis of Nicotiana benthamiana berberine bridge‐like (NbBBL) genes. Table S2 Primers used for amplification of sgRNA scaffolds. Table S3 Primers used for construction of the mobile single guide RNA plasmid vectors. Table S4 Primers used for genotyping Nicotiana benthamiana plants with Cas9‐induced mutations. Table S5 Comparison of Nicotiana benthamiana genome assemblies. Table S6 NbBBLa genotypes in Nicotiana benthamiana plants with Cas9‐mediated mutations. Table S7 NbBBLb genotypes in Nicotiana benthamiana plants with Cas9‐mediated mutations. Table S8 NbBBLc genotypes in Nicotiana benthamiana plants with Cas9‐mediated mutations. Table S9 NbBBLd genotypes in Nicotiana benthamiana plants with Cas9‐mediated mutations. Table S10 NbBBLd′ genotypes in Nicotiana benthamiana plants with Cas9‐mediated mutations. Table S11 Adjusted P‐values (single‐step method) of ANOVA and post hoc Tukey tests for Fig. 4. Table S12 Adjusted P‐values (single‐step method) of ANOVA and post hoc Tukey tests for Fig. 6. Please note: Wiley is not responsible for the content or functionality of any Supporting Information supplied by the authors. Any queries (other than missing material) should be directed to the New Phytologist Central Office. [file NPH-240-302-s001.pdf]

New Phytologist

***New Phytologist* Supporting Information**

Article title: An improved Nicotiana benthamiana bioproduction chassis provides novel insights into nicotine biosynthesis.

Authors: Katharina Vollheyde, Quentin M. Dudley, Ting Yang, Mehmet T. Oz , Davide Mancinotti, Mariano Olivera Fedi, Darren Heavens, Gareth Linsmith, Monika Chhetry, Mark A. Smedley, Wendy A. Harwood, David Swarbreck, Fernando Geu-Flores, Nicola J. Patron.

Article acceptance date: 28th June 2023.

The following Supporting Information is available for this article:

Fig. S1 Schematic and photographs of the hydroponic system.
Fig. S2 Maximum likelihood tree and multiple sequence alignment of *Nicotiana tabacum* and *Nicotiana benthamiana BBL* genes.
Fig. S3 Analysis of (*S*)- and (*R*)-nicotine in leaves of the quintuple *NbBBL* mutant (line 102) in comparison to control lines (WT and Cas9).
Fig. S4 Dihydrometanicotine (DMN) accumulation in roots of the quintuple *NbBBL* mutant (line 102) in comparison to two control lines (WT and Cas9), as analyzed by LC-MS.
Table S1 Primers used for expression analysis of *Nicotiana benthamiana berberine-bridge like* (*NbBBL*) genes.
Table S2 Primers used for amplification of sgRNA scaffolds.
Table S3 Primers used for construction of the mobile single guide RNA plasmid vectors.
Table S4 Primers used for genotyping *Nicotiana benthamiana* plants with Cas9-induced mutations.
Table S5 Comparison of *Nicotiana benthamiana* genome assemblies.
Table S6 NbBBLa genotypes in *Nicotiana benthamiana* plants with Cas9-mediated mutations.
Table S7 NbBBLb genotypes in *Nicotiana benthamiana* plants with Cas9-mediated mutations.
Table S8 NbBBLc genotypes in *Nicotiana benthamiana* plants with Cas9-mediated mutations.
Table S9 NbBBLd genotypes in *Nicotiana benthamiana* plants with Cas9-mediated mutations.
Table S10 NbBBLd' genotypes in *Nicotiana benthamiana* plants with Cas9-mediated mutations.
Table S11 Adjusted *p*-values (single-step method) of ANOVA and post hoc Tukey tests for Figure 4.
Table S12 Adjusted *p*-values (single-step method) of ANOVA and post hoc Tukey tests for Figure 6.
Methods S1 Genome sequencing and assembly.
Methods S2 Liquid chromatography–mass spectrometry (LC–MS) analysis of methanolic extracts.

Fig. S1 Schematic and photographs of the hydroponic system. For precursor feeding experiments and root harvesting, *Nicotiana benthamiana* seedlings were germinated and grown on cotton gauze (28 thread) glued onto rubber O-rings (¾ inch or 1 ¼ inch) fitted onto the wells of multi-well plates (6 or 12 wells, respectively).

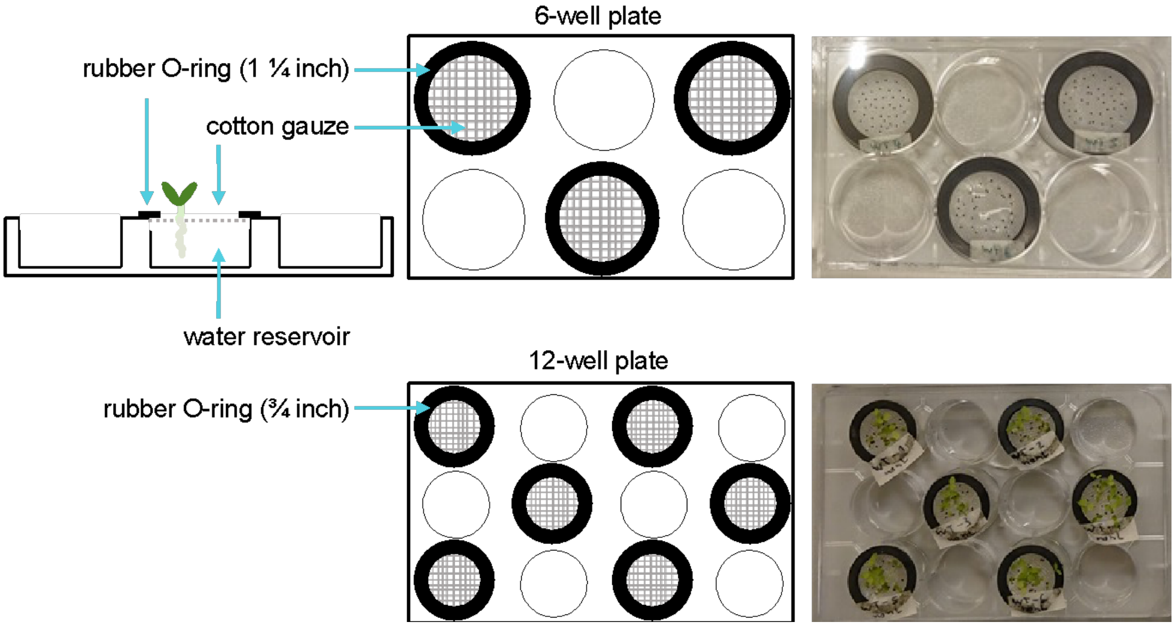

Fig. S2 Maximum likelihood tree and multiple sequence alignment of *Nicotiana tabacum* and *Nicotiana benthamiana* BBLs. The phylogeny was reconstructed using PhyML3.0. The premature stop codon in NbBBLd' is indicated by red, capitalized text. Scale bar indicates number of substitutions per site. Grey circles indicate boot strap support >90%.

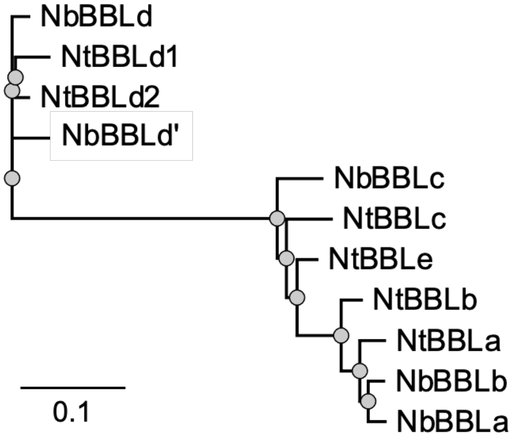


```
Consensus atg---------------------------tttccgctcataattct------gatcagc    27
NbBBLc    atg---------------------------tttctactcataattct------gatcagc    27
NbBBLa    atg---------------------------tttccgctcataattct------catcagc    27
NbBBLb    atg---------------------------tttccactcataattct------gatcagc    27
NtBBLc    atg---------------------------tttccactcataattct------aatcagc    27
NtBBLe    atg---------------------------tttccaatcataattct------gatcagc    27
NtBBLa    atg---------------------------tttccgctcataattct------gatcagc    27
NtBBLb    atg---------------------------tttccactcataattct------gatcagc    27
NbBBLd    atgaaacgaaatatatccatgtttcttcagcttctgctcataattctgatgatgatcagc    60
NbBBLd’   atgaagcgaaatatatccatgtttcttcagcttctgctcataattctaatgatgatcagc    60
NtBBLd1   atgaaacgaaatatatccatgtttcttcagcttctgctcattattctgatgatgatcagc    60
NtBBLd2   atgaaacgaaatatatccatgtctcttcagcgtttgctcataattctgatgatgatcagc    60

Consensus ttttcatttacttccctctctgctactgcta------ctagtggagca---g---gagga    75
NbBBLc    ttttcacttacttcccgctctgctactgctactgctactagtggagca---g---aagga    81
NbBBLa    ttttccc---------tctctgctactgcta------ctagtggagca---g---gagga    66
NbBBLb    ttttcacttacttccctctctgctactgcta------ctagtggagca---gaaggagga    78
NtBBLc    ttctcatttactttcctctctgctagtgcta------ctagtggagca---ggagaagga    78
NtBBLe    ttttcatttactttcctctttgctagtgtta------ctagtggagca---g---gagga    75
NtBBLa    ttttcacttgcttccttgtctgaaactgcta------ct------------g---gagct    66
NtBBLb    ttttcacttacttccctctctgctactgcaa------ctagtggagcaggag---gtgga    78
NbBBLd    ttcttatttacttctcttcttgtaccatccg------tctct---gca---a---caaat   105
NbBBLd’   ttcttatttacttgtcttcttgtatcttcca------tctct---gca---a---caaat   105
NtBBLd1   ttcttatttacttctcttcttgtaccttcgg------tctct---gca---a---caact   105
NtBBLd2   ttcttatttacttctcttcttgtaccttccg------tctct---gct---a---caaat   105

Consensus gttacaaatctttccacctgtttaatcaaccacaatgtccataacttctctatttacccc   135
NbBBLc    gttacaaatctttccacctgtttaatcaaccacaatgtccataacttctctatataccccc  141
NbBBLa    gttacaaatctttcaacctgtttaatcaaccacaatgtccataacttctctatttacccc   126
NbBBLb    gttacaaatctttcaacctgtttaaccaaccacaatgtctataacttctctatttacccc   138
NtBBLc    gttgcaaatctttccacctgtttaatcaaccacaatgtccataatttctccatgtacccc   138
NtBBLe    gttacaaatctttccacctgtttaatcaaccacaatgtccataacttctctatttacccc   135
NtBBLa    gttacaaatctttcagcctgcttaatcaaccacaatgtccataacttctctatttacccc   126
NtBBLb    gttgcaaatctttacacctgtttaatcgaccacaatgtccataacttctctatttacccc   138
NbBBLd    ctcaataccatttccacctgtttattcaattataaagtcactaacttctctgtttaccca   165
NbBBLd’   ctcaataccatttccacctgtttaatcaattacaaagtcagtaacttttctgtttaccca   165
NtBBLd1   ctcaataccatttccacctgtttaatcaattacaaagtcagtaacttctctgtttaccca   165
NtBBLd2   ctcaataccatttccacctgtttgatcaattacaaagtcagtaacttctctgtttatcca   165

Consensus acaaggaatgatccaa---gtagtaattactttaacttgctcgatttctcccttcagaat   192
NbBBLc    acaaagaatgatccaa---gtagaaattactttaacttgctcgatttctcccttcaaaat   198
```

```
NbBBLa    acaaggaacgatccaa---atagtaattactttaacttgctcgacttctcccttcagaat   183
NbBBLb    agaaggaatgatccaa---atagttcttactttaacttgctcgacttctcccttcagaat   195
NtBBLc    acaag---------------tagaaattactttaacttgctcgacttctcccttcagaat   183
NtBBLe    acaaagaatgatcaaagtagtagtaattactttaacttgctcgatttttcccttcagaat   195
NtBBLa    acaag---------------tagaaattactttaacttgctccacttctcccttcaaaat   171
NtBBLb    acaaagaatgatcaaagtagtagtaattactttaacttgctcgatttttcccttcagaat   198
NbBBLd    acaaggaatcatgctg---gtaatagttactataacttgcttgatttctccattcagaat   222
NbBBLd’   acaaggaatcatgctg---ataatagttactacaacttgcttgatttctccattcagaat   222
NtBBLd1   acaaggaatcatgctg---gtaatagttactataacttgcttgatttctccattcagaat   222
NtBBLd2   acaaggaatcatgctg---gtaataggtactataacttgcttgatttctccattcagaat   222

Consensus cttcgatttgcagcatcttacatgccgaaaccaacggtcattatcctaccaaacagcaag   252
NbBBLc    cttcgatttgctgcatcttacatgccgaaaccaacggtcattatcctaccaagcagcaag   258
NbBBLa    cttcgatttgctgcatcttacatgccgaaaccaaccttcattatcctaccaagcagcaag   243
NbBBLb    cttcgatttgcagcatcttacatgccgaaaccaacgttcattatcctaccaagcagcaag   255
NtBBLc    cttcgatttgcagcatctaacatgccgaaaccaacggtcattatcctaccaaacagcaag   243
NtBBLe    cttcgatttgctgcatcttacatgccgaaaccaacggtcattatcctaccaaacagcaaa   255
NtBBLa    cttcgctttgctgcacctttcatgccgaaaccaaccttcattatcctaccaagcagtaag   231
NtBBLb    cttcgatttgctgcatcttacatgccgaaaccaacggtcattattctaccaaacagcaaa   258
NbBBLd    ctccgattcgcagcgtcctctaaaccaaaaccaacggtcattattgtaccagagagcaag   282
NbBBLd’   ctcagattcgcagcgtcctctaatccaaagccaactgttattattgtaccagagatcaag   282
NtBBLd1   ctccgattcgcagcgtgctctaaaccaaaaccaactgtcattatcgtaccagagagcaag   282
NtBBLd2   ctccgattcgcagcgtcctctaaaccaaaaccaacggtcattatcgtaccagagagcaag   282

Consensus gaggagctcgtgagcaccattctttgttgcagacaagcatcttatgaaatcagagtaagg   312
NbBBLc    gaggagctcgtgagcaccattttctgttgcagaaaagcatcttatgaaatcagagtaagg   318
NbBBLa    gaggagctcgtgagcaccatttttttgttgcagaaaagcatcttatgaaatcagagtaagg   303
NbBBLb    gaggagctcgtgagcaccatttttttgttgcagaaaagcatcttatgaaatcagagtaagg   315
NtBBLc    gaggagctcgtgagcaccattctttgttgcagacaaacatcttatgaaatcagagtaagg   303
NtBBLe    gaggagctcgtgagtaccattctttgttgcagacaaacatcttatgaaatcagagtaagg   315
NtBBLa    gaggagctcgtgagcaccatttttttgttgcagaaaagcatcttatgaaatcagagtaagg   291
NtBBLb    gaggagctcgtgagcaccattctttgttgcagacaagcttcttatgaaatcagagtaagg   318
NbBBLd    gagcagctggtgagcagcgttctgtgttgcagacaaggttcttatgaaatcagagtaagg   342
NbBBLd’   gagcagctggtgagcagcgttctgtgttgcagacaaggttcttatgaaatcagagtaagg   342
NtBBLd1   gagcagctggtgagcagcgttctgtgttgcagacaaggctcgtatgaaatcagagtaagg   342
NtBBLd2   gagcagctggtgagcagcgttctgtgttgcagacaaggttcttatgaaatcagagtaagg   342

Consensus tgcggaggacacagttacgaaggaacttcttacgtttcctttgacggttccccattcgtg   372
NbBBLc    tgcggaggacacagttatgaaggaacttcttacgtttcctttgacggttctcaattcgtg   378
NbBBLa    tgcggcggacacagttacgagggaacatcttacgtttcctttgatgcttccccccttcgtg   363
NbBBLb    tgcggcggacacagttacgagggaacatcttacgtttcctttgacggttccccgttcgtg   375
NtBBLc    tgcggaggacacagttacgaaggaacttcttctgtttcctttgacggttccccgttcgtg   363
NtBBLe    tgcggaggacacagttacgagggaacttcttacgtttcctttgacggttcccctttcgtg   375
NtBBLa    tgcggcggacacagttacgaaggaacttcttacgtttcctttgacgcttctccattcgtg   351
NtBBLb    tgcggaggacatagttacgagggaacttcttacgtttcctttgacggttccccgttcgtg   378
NbBBLd    tgcggaggacacagttatgaaggaacttcttacgtttcctttgatggttccccatttgtg   402
NbBBLd’   tgcggaggacacagttatgaagggacttcatacgtttcctttgatggttccccatttgtt   402
NtBBLd1   tgcggtggacacagttatgaagggacttcatcagtttcctttgatggttccccatttgtg   402
NtBBLd2   tgcggaggacacagttatgaagggacttcttacgtttcctttgatggttccccatttgtg   402

Consensus atcgttgacttgatgaaattagacgacgtttcagtagatttggattccgaaacagcttgg   432
NbBBLc    atcgttgacttgatgaaattagacgacgtttcagtagatttggattccgaaacagcttgg   438
NbBBLa    atcgttgacttgatgaaattagacaacgtttcagtagatttggattccgaaactgcttgg   423
NbBBLb    atcgttgacttgatgaaattagacgacgtttcagtagatttggattctgaaactgcttgg   435
NtBBLc    atcatcgacttgatgaaattagacgacgtttcagtagatttggattccgaaactgcttgg   423
NtBBLe    atcgttgacttgatgaaattagacgacgtttcagtagatttggattccgaaacagcttgg   435
NtBBLa    atcgttgacttgatgaaattagacgacgtttcagtagatttggattctgaaacagcttgg   411
NtBBLb    atcgttgacttgatgaaattagacgaagtttcagtagatttggattctgaaactgcttgg   438
NbBBLd    gtaattgatttaatgaaattagacggcatttcagtagatttggattccgaaacagcttgg   462
NbBBLd’   gtcattgatttgatgaaattagacggcgtttcagtagatttggattccgaaacagcaTGA   462
NtBBLd1   gtcattgatttgatgaaattagacggcgtttcagtggatgtggattcagaaaccgcgtgg   462
NtBBLd2   gtcattgatttgatgaaattagatgatgtttcggtagatttggattccgaaaccgcgtgg   462

Consensus gctcagggcggcgcaacaattggccaaatttattacgccattgccaaggtaagtgacgtt   492
NbBBLc    gcccagggcggcgcaacaattggccaaatttattacgccattgccaaggtaagtgatgtt   498
NbBBLa    gcccagggcggcgcaacaattggccaaatttattacgccattgccaaggtaagtgacgtt   483
NbBBLb    gctcagggaggcgcaacaattggccaaatttattacgccattgccaaggcaagtgacgtt   495
NtBBLc    gctcagggcggcgcaacaattggccaaatttattacgccattgccaaggcaagtgacgtt   483
NtBBLe    gctcagggcggcgcaacaattggccaaatttattacgccatttccagggttagtgacgtt   495
NtBBLa    gctcagggcggcgcaacaattggccaaatttattatgccattgccaaggtaagtgacgtt   471
NtBBLb    gctcagggcggcgcaacaattggccaaatttattacgccattgccaaggtaagtgacgtt   498
```

```
NbBBLd    gtacaaggtggcgctacacttggccagacttattatgccatttcccaggccagcgacgtt  522
NbBBLd’   gtacaaggtggcactacacttggccagacttattatgccatttcccaggtcagtggcgtt  522
NtBBLd1   gtacagggcggcgctacacttggccagacttattatgccatttcccgagccagcaacgtt  522
NtBBLd2   gtacaaggtggcgctacacttggccagacttattatgccatttcccgggccagtgacgtt  522

Consensus catgcattttcagcaggttcgggaccaacagtaggatctggaggtcatatttcaggtggc  552
NbBBLc    catgcattttcagcaggttcggcatcaacagtaggatctggcggtcatatttcaggtggc  558
NbBBLa    catgcattttcagcaggttcgggaccaaccgtaggatctggaggtcatatttccggtggc  543
NbBBLb    catgcattttcagcgggttcggggccaacagtaggatctggaggtcatatttccggtggc  555
NtBBLc    catgcattttcagcaggttcgggtccaacagtaggatctggaggtcatatttcaggtggc  543
NtBBLe    catgcattttcagcaggttcgggaccaacagtaggatctggaggtcatatttcaggtggc  555
NtBBLa    catgcattttcagcaggttcgggaccaacagtaggatctggaggtcatatttcaggtggt  531
NtBBLb    catgcattttcagcaggttcgggaccaacagtaggatctggaggtcatatttcaggtggc  558
NbBBLd    catggattttcagctggttcttgcccaacagttggggttgggggccacatttccgggggt  582
NbBBLd’   catggattttcagctggttcttgcccaacagttggggtcgggggccacatttcagggggt  582
NtBBLd1   catggattttcagctggttcttgcccaacagttggggttggcgggcacatttccgggggt  582
NtBBLd2   catggattttcagctggttcttgcccaacagttggggttgggggccacatttccgggggt  582

Consensus ggctttggacttttatcyagaaaattcggacttgctgctgataatgtcgttgatgctctt  612
NbBBLc    ggctttggacttttgtccagaaaattcggactcgctgcggataatgttgttgatgctctt  618
NbBBLa    ggatttggacttttatctagaaaattcggacttgctgctgataatgtcgttgatgctctt  603
NbBBLb    ggatttggacttttatctaggaaattcggacttgctgctgataatgtcgttgatgctctt  615
NtBBLc    ggctttggacttctgtccagaaaattcggagtcgctgctgatagtgtcgttgatgctctt  603
NtBBLe    ggctttggactaatgtccagaaaattcggactcgctgctgatagtgtcgttgatgctctt  615
NtBBLa    ggatttggacttttatctagaaaattcggacttgctgctgataatgtcgttgatgctctt  591
NtBBLb    ggctttggacttttatctagaaaattcggactcgctgctgataatgtcgttgatgctctt  618
NbBBLd    ggctttggatttttatcaagaaaatatggacttgctgctgataacgtggtcgatgctctt  642
NbBBLd’   ggctttggatttttgtcaaggaaatatggacttgctgctgataacgtggtggatgctctt  642
NtBBLd1   ggttacggatttttatccagaaaatatggacttgctgctgataacgtggtggatgctctt  642
NtBBLd2   ggctttggatttttatcaagaaaatatggacttgctgctgataacgtggtggatgctctt  642

Consensus cttattgatgctgaaggacggttattagaccgaaaagccatgggagaagacgtgttttgg  672
NbBBLc    cttattgatgctgaaggacggttattagaccgaaaagccatgggagaggacgtattttgg  678
NbBBLa    cttattgatgctgatggacggttattagaccgaaaagccatgggtgaagacgtgttttgg  663
NbBBLb    ctaattgatgctgatggacggttattagaccgaaaagccatgggagaagacgtgttttgg  675
NtBBLc    cttattgatgctgatggacggttattagaccgaaaagccatgggagaagacgtgttttgg  663
NtBBLe    ctaattgatgctgaaggacggttattagaccggaaagccatgggagaagacgtattttgg  675
NtBBLa    cttattgatgctgatggacggttattagaccgaaaagccatgggcgaagacgtgttttgg  651
NtBBLb    cttatcgatgctgatgggcggttattagaccgaaaagccatgggagaagacgtgttttgg  678
NbBBLd    cttgttgattcggaaggacggctattagaccgcaaagccatgggagaagaagtgttttgg  702
NbBBLd’   cttgttgatgcggaaggacggctattagaccgctaagccatgggagaagaagtgttttgg  702
NtBBLd1   cttgttgatgcggaaggacggctattagaccgcaaagccatgggagaagaaatcttttgg  702
NtBBLd2   cttgttgatgcggaaggacggctattagaccgcaaagccatgggagaagaagtgttttgg  702

Consensus gcaatcagaggtggcggyggtggaaattggggaattatttatgcctggaaaattcgatta  732
NbBBLc    gcaatcagaggtggaggcggtggaaattggggaataatttatgcctggaaaatcagatta  738
NbBBLa    gcaatcagaggtggcggtggtggaaattggggaattgtttatgcctggaaaattcgatta  723
NbBBLb    gcaatcagaggtggcggcggtggaaattggggaattgtttatgcctggaaaattcgatta  735
NtBBLc    gcaatcagaggtggcggcggtggaaattggggaattatttatgcctggaaaattcgatta  723
NtBBLe    gcaatcagaggtggcggcggtggaaattggggaattatttatgcctggaaaattcgatta  735
NtBBLa    gcaatcagaggtggcggcggtggaaattggggcattgtttatgcctggaaaattcgatta  711
NtBBLb    gcaatcagaggtggcggaggaggaaattggggaattatttatgcctggaaaattcgatta  738
NbBBLd    gccatcagaggtggtggtggaggaatttggggaatcatttatgcctggaaaatccgattg  762
NbBBLd’   gccatcagaggtggtggtggagggatttggggaatcatttacgcctggaaaatccgattg  762
NtBBLd1   gccatcagaggtggaggtggaggaatttggggaatcatttacgcctggaaaatccgattg  762
NtBBLd2   gccatcagaggtggtggtggaggaatttggggaatcatttacgcctggaaaatccgattg  762

Consensus ctcaaagtgcctaaaatcgtaacaacttttatgatctctaggcctggmtccaaacaatac  792
NbBBLc    atcaaagtgcctaaaatcgtaacaacttttgtgatctctaggcctggctccaaacaatac  798
NbBBLa    ctcaaagtgcctaaaattgtaacagcttgtatgatctataggcctggatccaaacaatac  783
NbBBLb    ctcagagtgcctaaaatcgtaacagcttgtatgatctataggcctggatccaaacaatac  795
NtBBLc    gtgaaagtgcccaaaatcgtaacaacttttaagatctctaagcctggctccaaacaatac  783
NtBBLe    ctcaaagtgcctaaaatcgtaacaacttgtatgatctataggcctggatccaaacaatac  795
NtBBLa    ctcaaagtgcctaaaatcgtaacaacttgtatgatctataggcctggatccaaacaatac  771
NtBBLb    ctcaaagtgcctaaaatcgtaacaacttgtatgatctataggcctggatccaaacaatac  798
NbBBLd    ctcaaagtgcccaagaccgtgactagtttcatagtccctaggcctggctccagacgatat  822
NbBBLd’   ctcaaagtgcccaagactgtgactagtttcataatccctaggcctgggtccaaacgat--  820
NtBBLd1   ctcaaagtgcccaagaccgtgaccagtttcataatccctaggcctggctccaaacgatat  822
NtBBLd2   ctcaaagtgcccaagactgtgactagtttcatagtccctaggcctggctccaaacgatat  822

Consensus gtggcccaactacttcacaaatggcaaatagttgcaccaaatttggacgatgattttact  852
```

```
NbBBLc    gttgcctcattacttcacaaatggcaattagttgcaccaaatttggacgatgattttact   858
NbBBLa    gtggctcaaatacttgagaaatggcaagtagttactccaaatttagtcgatgattttact   843
NbBBLb    gtggctcaaatacttgagaaatggcaaattgttactccaaatttggtcgatgattttact   855
NtBBLc    gttgccccattactttacaaatggcaaatagttgcaccaaatttggccgatgattttact   843
NtBBLe    gtggctcaactacttcagaaatggcaaatagttactccaaatttggccgatgattttact   855
NtBBLa    gtggctcaaatacttgagaaatggcaaatagttactccaaatttggtcgatgattttact   831
NtBBLb    gtggctcaactacttcagaaatggcaaatagttactccaaatttggtcgatgattttact   858
NbBBLd    gtgtcccaactagttcacaaatggcaacttgttgcaccaaagttagacgatggcttttat   882
NbBBLd’   gtgtcccaactagttcacaaatggcaacttgttgcaccaaagttagacgatgacttttat   880
NtBBLd1   gtgtcccaactagttcacaaatggcaacttgttgcaccaaagttagaggatgaattttat   882
NtBBLd2   gtgtcccaactagttcacaaatggcaacttgttgcaccaaagttagacgatgacttttat   882

Consensus ctaggagtactcatgagacctgcaaatctnccggcggatatgaaatawgg-aatamtact   911
NbBBLc    ctaggagtagggatgggaccattcaatctgccagcggatatgaaatacggaaatactact   918
NbBBLa    ctaggagtactcctgaggcctgcagatctaccggcggatatgaaatatggtaatagtact   903
NbBBLb    ctaggagtactcctgagacctgcaaatctaccggcggatatgaaatatggtaatagtact   915
NtBBLc    ctaggagtacaaatgatacctatagatctgccggctgatatgaaatacggaaatcctact   903
NtBBLe    ctaggagtactcatgagacctatagatctgcgggcggatatgaattacggaaatactact   915
NtBBLa    ctaggagtactgctgagacctgcagatctacccgcggatatgaaatatggtaatactact   891
NtBBLb    ctaggagtactcttgagacctgcagatctaccggcggatatgaaatatggcaacagtact   918
NbBBLd    ctatcgatctccatgagctctgctagtaa------------------agg---aaacatt   921
NbBBLd’   atatcgatctccatgagctctgctagtaa------------------agg---aaacatt   919
NtBBLd1   ctatcgatctccatgagctctcctagtaa------------------agg---aaacatt   921
NtBBLd2   ctatcgatctccatgagctctgctagtaa------------------agg---aaacatt   921

Consensus cctattgaaatatttccccaattcaacgcactttatttgggtccaaaaactgaagccatt   971
NbBBLc    cctgttgaaataattccccaattcaacgcactttatttgggtccaaaaactgaagctatt   978
NbBBLa    cctattgaaatatttccccaattcaacgcactttatttgggtccaaaaactgaagttctt   963
NbBBLb    cctattgaaatatttccccaattcaacgcgctttatttgggtccaaaaactgaagctctt   975
NtBBLc    cctattgaaatatgtccccaattcaatggactttatctgggtccaaaaactgaagcggtt   963
NtBBLe    cctattgaaacatttccccaattcaatgcactttatttgggtccaaaaactgaagcggtt   975
NtBBLa    cctattgaaatatttcccaattcaatgcactttatttgggtccaaaaactgaagttctt   951
NtBBLb    cctattgaaatatttccccaattcaatgcactttatttgggtccaaaaactgaagtcctt   978
NbBBLd    cctattgaagtaaatgcccaattcagcggattttaccttggtacaaaaaccgaagccatt   981
NbBBLd’   ccttttgaaataaatgcccaattcagcggattttacttaggtacaaaaaccgaagccatt   979
NtBBLd1   cctattgaaataaatgcccaattcagcggattttacctaggtacaaaaaccgaagccatt   981
NtBBLd2   cctattgaaataaatgcccaattcagcggattttacctaggtacaaaaaccgaagccatt   981

Consensus tccatattgaatgaggcatttccggagctgggcgttaagaatgatgactgcaaagaaatg  1031
NbBBLc    tccatattaaatgaggcatttccagagctggacgttaagaatgatgacggcaaagaaatg  1038
NbBBLa    tccatatcaaatgagacatttccggagctaggcgttaagagtgatgagtgcaaggaaatg  1023
NbBBLb    tccatatcaaatgagacatttccggagctaggcgttaagaatgacgagtgcacggaaatg  1035
NtBBLc    tctatattaaatgaggcatttccagagctgaacgttaagaatgatgacgccaaagaaatg  1023
NtBBLe    tccatattaaatgaggcatttccagagctggacgctaagaatgatgacgccaaagaaatg  1035
NtBBLa    tccatatcgaatgagacatttccggagctaggcgttaagaatgatgagtgcaaggaaatg  1011
NtBBLb    tctatatcgaatgaggaatttccggagctgggcgttaagaatgatgagtgcaaggaaatg  1038
NbBBLd    tccatcttgaatgaggcctttccggaggtcggagttctggaagatgactgcaaagaaatg  1041
NbBBLd’   tccatcttgaacgaggcctttccggaggtgggagttctggaagatgactgcatagaaatg  1039
NtBBLd1   tccatcttgaatgaggccttttcggagttgggagttctggaaggtgactgcaaagaaatg  1041
NtBBLd2   tccatcttgaatgaggcctttccggagttgggagttgtggaaagtgactgcaaagaaatg  1041

Consensus acttggattgagtcagcacttttcttctccgaattagataacgtta-cgggaactcctct  1090
NbBBLc    acttggatagagtcagcacttatcttttccgaatcagctaacgtattcgggaactcttcc  1098
NbBBLa    acttgggtagagtcatcactttacttctccgaattagctgacgttagcgggaactcctct  1083
NbBBLb    acttgggtagagtcatcactttacttctccgaattagctgacgttagcgggaactcctct  1095
NtBBLc    acttggattgagtctgcacttttcttttccgacttagataacatattcgggaactcctct  1083
NtBBLe    acttggattgagtcagcacttttcttttccgaattagataacgtattcgggaactcctct  1095
NtBBLa    acttgggtagagtcagcacttttcttctccgaattagctgacgttaacgggaactcgact  1071
NtBBLb    acttggatagagtcagcacttttcttctccgaattagctgacattaacgggaattcctct  1098
NbBBLd    agttggattgaatcaacacttttcttctccgaattagataacgttg---cgaacacctc-  1097
NbBBLd’   agttggattgaatcaacgcttttcttctccaaattagataacggta---cgaacacctc-  1095
NtBBLd1   agttggattgaatcaacacttttcttctccgaattaaatgacgttg---cgaattcctc-  1097
NtBBLd2   agttggattgaatcaacacttttcttctccgaattagataacgttg---cgaacacctc-  1097

Consensus gncgatatctcccgtttgaaagaacgttacatggacggaaaatctttcttcaaaggcaaa  1150
NbBBLc    gacgatatctcccgtttgagagaacgctacacggacggaaaatctttcttcaaatgcaaa  1158
NbBBLa    ggtgatatctcccgtctgaaagaacgttacatggacggaaaaggtttcttcaaaggcaag  1143
NbBBLb    gctgatatctcccgtctgaaagaacgttacatggacggaaaaggtttcttcaaaggcaag  1155
NtBBLc    gacgatatctcccatttgaaagaacgctacttgggtgtaaaaatttgcttcaaaggcaaa  1143
NtBBLe    gacgatatctcccgtttgaaagaacgctacatggacgcaaaaactttcttcaaaggcaaa  1155
NtBBLa    ggtgatatctcccgtctgaaagaacgttacatggacggaaaaggttttttcaaaggcaaa  1131
```

```
NtBBLb    aatgatatctcccgtctgaaagaacgttacatggacggaaaaggtttcttcaaaggcaaa  1158
NbBBLd    --cgatgtctctcgtctaaaagaacgttactttgaaaacaaatcatacttcaaagccaaa  1155
NbBBLd’   --cgatgtctctcgtttgaaagagcgttactttgaaaaccaatcatacttcaaagccaaa  1153
NtBBLd1   --cgatgtctctcgtttgaaagagcgttactttgaaaacaaatcatacttcaaagccaaa  1155
NtBBLd2   --cgatgtctctcgtttgaaagagcgttactttgaaaacaaatcatacttcaaagccaaa  1155

Consensus tcagactatgtgaagaccccartttcaatggatgggatgatgacagctcttgttgaactc  1210
NbBBLc    tcagattatgtgaagatcccattttcaatggacgggatgatgacagctcttgttgaacta  1218
NbBBLa    acggactatgtgaagaagccagtttcaatggatgggatgctcacatttcttgttgaactc  1203
NbBBLb    acggactatgtgaagaagccagtttcaatggatgggatgctcacatttcttgttgaactc  1215
NtBBLc    tcagattatgtgaagaccccattttcaatggacggaataatgacagctcttgttgaacac  1203
NtBBLe    tcagattttgtgaagactccattttcaatggacgcgatgatgacagctcttgttgaactc  1215
NtBBLa    acggactacgtgaagaagccagtttcaatggatgggatgctaacatttcttgtggaactc  1191
NtBBLb    acggactatgtgaagaagccagtttcaatggatgggatgctaacatttcttgttgaactc  1218
NbBBLd    tcagactatgtgaagaccccaatttcagtgggagggattatgacagctcttgatgttctt  1215
NbBBLd’   tcagactatgtgaagaccccaatttcagtgggagggattatgacagctcttgatgttctt  1213
NtBBLd1   tcagactatgtgaagaccccaatttcagtgggtgggattatgacggctcttaatgttctt  1215
NtBBLd2   tcagaccatgtgaagaccccaatttcagtgggagggattatgacagctcttgatgttctt  1215

Consensus gagaaaaaccccaagggatatcttgtctttygatccttatggcggagccatggacaagatt  1270
NbBBLc    gagaaaaaacccaactcataccttatcttcgatccttatggtggagccatggacaagatt  1278
NbBBLa    gagaaaaacccgaagggataccttgtcttcgatccttatggcggagccatggacaagatt  1263
NbBBLb    gagaaaaacccgaagggataccttgtcttcgatccttatggcggagccatggacaagatt  1275
NtBBLc    gagaaaaaccccaatgcattccttgtgttcgatccatatggcggagccatggacaaaatt  1263
NtBBLe    gagaaaaaccccaagtcattccttgtcttcgatccttatggcggagtcatggacaagatt  1275
NtBBLa    gagaaaaacccgaagggatatcttgtctttgatccttatggcggagccatggacaagatt  1251
NtBBLb    gagaaaaacccgaagggatatcttgtctttgatccatatggcggagccatggacaagatc  1278
NbBBLd    gagaaagaacccaatggacatgtcatatttgacccttatggtgcagccatgcagagaatt  1275
NbBBLd’   gagaaagaacc-aatggacatgttatctttgacccttatggtgcagccatgaagagaatt  1272
NtBBLd1   gagaaagaacccaacggacatgtcatcttggacccttatggtggagccatgcaaagaatt  1275
NtBBLd2   gagaaagaaccaaatggacatgtcatctttgacccttatggtgcagccatgcagagaatt  1275

Consensus agtgatcaagctattgctttccctcatagaaagggtaacctttttcgcrattcaatatcta  1330
NbBBLc    agtgatcaagctattcctttccctcatcgaaagagtaacctttttcgccgttcaatattat  1338
NbBBLa    agtgatcaagctattgctttccctcatagaaaaggtaacctttttcgcgattcagtatctg  1323
NbBBLb    agtgatcaagctattgctttccctcatagaaaaggtaacctttttcgcgattcagtatctg  1335
NtBBLc    agtgctcaagctattgctttccctcatcgaaagggtaacctttttcgcaattcaatattat  1323
NtBBLe    agtgatcaagctattgctttccctcatcgaaagggtaacctttttcgcggttcaatattat  1335
NtBBLa    agtgatcaagctattgctttccctcatagaaaaggtaacctttttcgcgattcagtatcta  1311
NtBBLb    gatgatcaagctattgcgttccctcatagaaagggtaatcttttcgcgattcaatatcta  1338
NbBBLd    agcgaggaagctattgctttccctcatagaaagggtaacctattcggaattcaatatcta  1335
NbBBLd’   ggcgaggaagctattgctttccctcatagaaagggtaatcttttcggaattcaatatcta  1332
NtBBLd1   agtgaggaagctattgctttccctcatagaaagggtaacctttttcggaattcaatatcta  1335
NtBBLd2   agcgaggaagctattgctttccctcatagaaagggtaacctattcagaattcaatatcta  1335

Consensus gcastgtggaawgaagaggacgatwa---------caagagcaa---cgkgtacatagag  1378
NbBBLc    gcagtgtggaacgaagaggacgatgc---------------cga---cgagtacttagag  1380
NbBBLa    gcacagtggaatgaagaggacgatta---------catgagcga---cgtttacatggag  1371
NbBBLb    gcacagtggaatgaagaggacgatta---------catgagcga---cgtttacatggag  1383
NtBBLc    gcacagtggaacgaagaggacgatgc---------caagagcaa---cgagcacatagag  1371
NtBBLe    gcattttggaacgaagaggacgatgc---------caagagcaa---cgagtacatagag  1383
NtBBLa    gcacagtggaatgaagaggacgatta---------catgagcga---cgtttacatggag  1359
NtBBLb    gcacagtggaatgaagaggacgatta---------caagagcga---tgtttacatggag  1386
NbBBLd    gtagtgtggaaagaaaaggacaataataatattgccaagagcaa---tgggtacatagag  1392
NbBBLd’   gtagtgtggaaagaaaaggacaataataatattgccaagaggaa---tggatacatagag  1389
NtBBLd1   gtagtgtggaaagaaaaggacaataataatattgtcaagagcaatattgggtacatagag  1395
NtBBLd2   gtagtgtggaaagaaaaggacaataataatattgccaagagcaa---tgggtacatagag  1392

Consensus tggataagaggattttacaatacaatggcgccctttgtttcaagctcgccaaggggagct  1438
NbBBLc    tggataagaggattttataataaaatggcgccttttgtttcaagctcgccaaggggagct  1440
NbBBLa    tggataagagggttttacaatacaatgaccccatttgtgtcaagctcgccaaggggagct  1431
NbBBLb    tggataagaggattttacaatacaatgacgccatttgtttcaagctcgccaaggggagct  1443
NtBBLc    tggataagaggattttacaataaaatggcgccttttgtttcaagctcgccaaggggagct  1431
NtBBLe    tggacaaggggattttacaataaaatggcgccttttgtttcaagctcgccaaggggagct  1443
NtBBLa    tggataagaggattttacaatacaatgacgccctttgtttcaagctcgccaaggggagct  1419
NtBBLb    tggataagaggattttacaatacaatgacgccctttgtttcaagctcgccaaggggagct  1446
NbBBLd    tggataagagagttttacaatacaatggcaccccatgtttcaacttcacctagggcagct  1452
NbBBLd’   tggataagagagctttacaatacaatggcaccccatgtttcaagttcaccaagggcagct  1449
NtBBLd1   tggataagagagttttacaatacaatggcaccccatgtttcaagttcacctagggcagct  1455
NtBBLd2   tggataagagagttttacaatacaatggcaccccatgtttctagttcacctagggcagct  1452
```

```
Consensus tatatcaactacttggatatggatcttggagtgaatatggtcgacgactacttaytgcga  1498
NbBBLc    tatatcaactacttggatatggatcttggagtgaacatggaccatgactacttactgcga  1500
NbBBLa    tatatcaactacttggatatggatcttggagtgaatatggtcgacaactacttattgcta  1491
NbBBLb    tatatcaactacttggatatggatcttggagtgaatatggtcgacaactacttattacga  1503
NtBBLc    tatgtcaactacttggatatggatcttggaatgaatatggacgacgactacttactgcga  1491
NtBBLe    tatatcaactacttggatatggatcttggagtgaatatggacgacgactacttactgcga  1503
NtBBLa    tatatcaactacttggatatggatcttggagtgaatatggtcgacgactacttattgcga  1479
NtBBLb    tatatcaactacttggatatggatcttggagtgaatatggacgacgactacttactgcga  1506
NbBBLd    tatgtcaactatatggatcttgaccttggagtga------tggacggctacttaatgcta  1506
NbBBLd'   tatgtcaactacatggatctggaccttacaa-----------------------------  1480
NtBBLd1   tatgtcaactacatggatctggaccttggagtga------tggacgactacttattgcca  1509
NtBBLd2   tatgtcaactatatggatctggaccttggagtga------tggacgactacttaatgcta  1506

Consensus aatgctagtagn---nnttcttcttcctctgttgatgctgtggagagagctagagcgtgg  1555
NbBBLc    aatacta------------cttcttcctctgttgatgctgtggagagagctagagcgtgg  1548
NbBBLa    aatgctagcagt---agttcttcttcctctgctgatgctgtggaaagagctagagcatgg  1548
NbBBLb    aatgctactagt---agttcttcttcctctgttgatgctgtggagagagctagagcgtgg  1560
NtBBLc    aatgctagtagt---cgttattcttcctctgttgatgctgtggagagggctagagcatgg  1548
NtBBLe    aatgctagtagtcgtagttcttcttcctctgttgatgctgtggagagagctagagcgtgg  1563
NtBBLa    aatgctagtagcagtagtccttcttcctctgttgatgctgtggagagagctagagcgtgg  1539
NtBBLb    aatgctagtagtcgtaattcttcttcctctgttgatgctgtggagagagctagagcgtgg  1566
NbBBLd    aatactagtac------tactgcctcttctgatcatgccgtggagagagcaagggtctgg  1560
NbBBLd'   ------------------------------------------------------------  1480
NtBBLd1   tgtactagtac------tactgcgtctgctaatcatgccgtggagagagcaagggtctgg  1563
NtBBLd2   aatactagtat------tactgcctctgctgatcatgccgtggagagagcaagggtctgg  1560

Consensus ggtgaaawgtatttcttgmataactatgataggttggttaaagctaagacacaaattgat  1615
NbBBLc    ggtgaaaagtatttcttgaacaactatgataggttggttaaagctaagacacaaattgat  1608
NbBBLa    ggtgaaatgtatttcttgcataactatgataggttggttaaagctaagacacaaattgat  1608
NbBBLb    ggtgaaatgtatttcttgcataactatgataggttggttaaagctaagacacaaattgat  1620
NtBBLc    ggtgaaaagtatttcttgaataactatgataggttggttaaagctaagacaaaaattgat  1608
NtBBLe    ggtgaaatgtatttcttgcataactatgataggttggttaaagctaagacacaaattgat  1623
NtBBLa    ggtgagatgtatttcttgcataactatgataggttggttaaagctaagacacaaattgat  1599
NtBBLb    ggtgaaatgtatttcttgcataactatgataggttggttaaagctaagacacaaattgat  1626
NbBBLd    ggtgaaaagtatttcttgaataactatgatagattggtcaaagctaagacaaaaattgac  1620
NbBBLd'   ------------------------------------------------------------  1480
NtBBLd1   ggtgaaaagtatttcttgaataactatgatagattggtcaaagctaagacaaaaattgac  1623
NtBBLd2   ggtgaaaagtatttcttgaataactatgatagattggtcaaagctaagacaaaaattgac  1620

Consensus ccactaaatgtttttcgacatgaacagagtattcctcctatgcttggttcaacgcaagag  1675
NbBBLc    ccactaaatgtttttcgacatgaacagagtattcctcctatgcttggttcaacgcaagac  1668
NbBBLa    ccactaaatgtttttcgacatgaacagagtattcctcctatgcgtggttcaacgcaagag  1668
NbBBLb    ccactaaatgttttccgccatgaacagagtattcctcctatgcttggttcaacgcaacag  1680
NtBBLc    ccactaaatgtttttcgacatgaacagagtatacctcctacgcttggttcaacgcaagag  1668
NtBBLe    ccactaaatgtttttcgacatgaacagagtatacctcctatgcttggttcaacgcaagag  1683
NtBBLa    ccactaaatgtttttcgacatgaacagagtattcctcctatgcttggttcaacgcaagag  1659
NtBBLb    ccacttaatgtttttcgacatgaacagagtatacctcctatgcttggttcaacgcaagag  1686
NbBBLd    ccactaaacgtttttcgacatcaacagggcatccctcctattttcgcctcaatgcaagag  1680
NbBBLd'   ------------------------------------------------------------  1480
NtBBLd1   ccactaaacgtttttcgacatcaacagggcatccctcctttgttcgcctcaatgcaagag  1683
NtBBLd2   ccactaaacgtttttcgacatcaacagggcatccctcctatgttcgcctcaatgccagag  1680

Consensus cataagtatagtagtgaatga                                         1696
NbBBLc    aataagtatagcagtgaatga                                         1689
NbBBLa    aacaagtatagtagcgaatga                                         1689
NbBBLb    cataagtatagtagtgaatga                                         1701
NtBBLc    cataactacagtagtgaatga                                         1689
NtBBLe    ca------cagtagtgaatga                                         1698
NtBBLa    cacaagtatagcagtgaatga                                         1680
NtBBLb    cataagtacagtagtgaatga                                         1707
NbBBLd    catacctacagtagtaaatga                                         1701
NbBBLd'   -------------ctacttaa                                         1488
NtBBLd1   tatacctatagtagtaaatga                                         1704
NtBBLd2   catacctatagtagtaaatga                                         1701
```

Fig. S3 Analysis of (*S*)- and (R)-nicotine in leaves of the quintuple *NbBBL* mutant (line 102) in comparison to control lines (WT and Cas9). Traces correspond to extracted ion chromatograms (nicotine, $[M+H]^+$) resulting from chiral LC-MS analyses (Lux® 3 µm AMP column). Traces on the upper half of the figure are from uninduced plants, while traces on the lower half are from the same plants 5 days after induction with MeJa. Higher injection volumes were used for all uninduced samples (10 µl compared to 2 µl) to obtain comparable peak sizes. A total of four to five biological replicates were analyzed. Traces on the left column show the results of running a racemic nicotine standard and a (*S*)-nicotine standard along with the uninduced and induced samples.

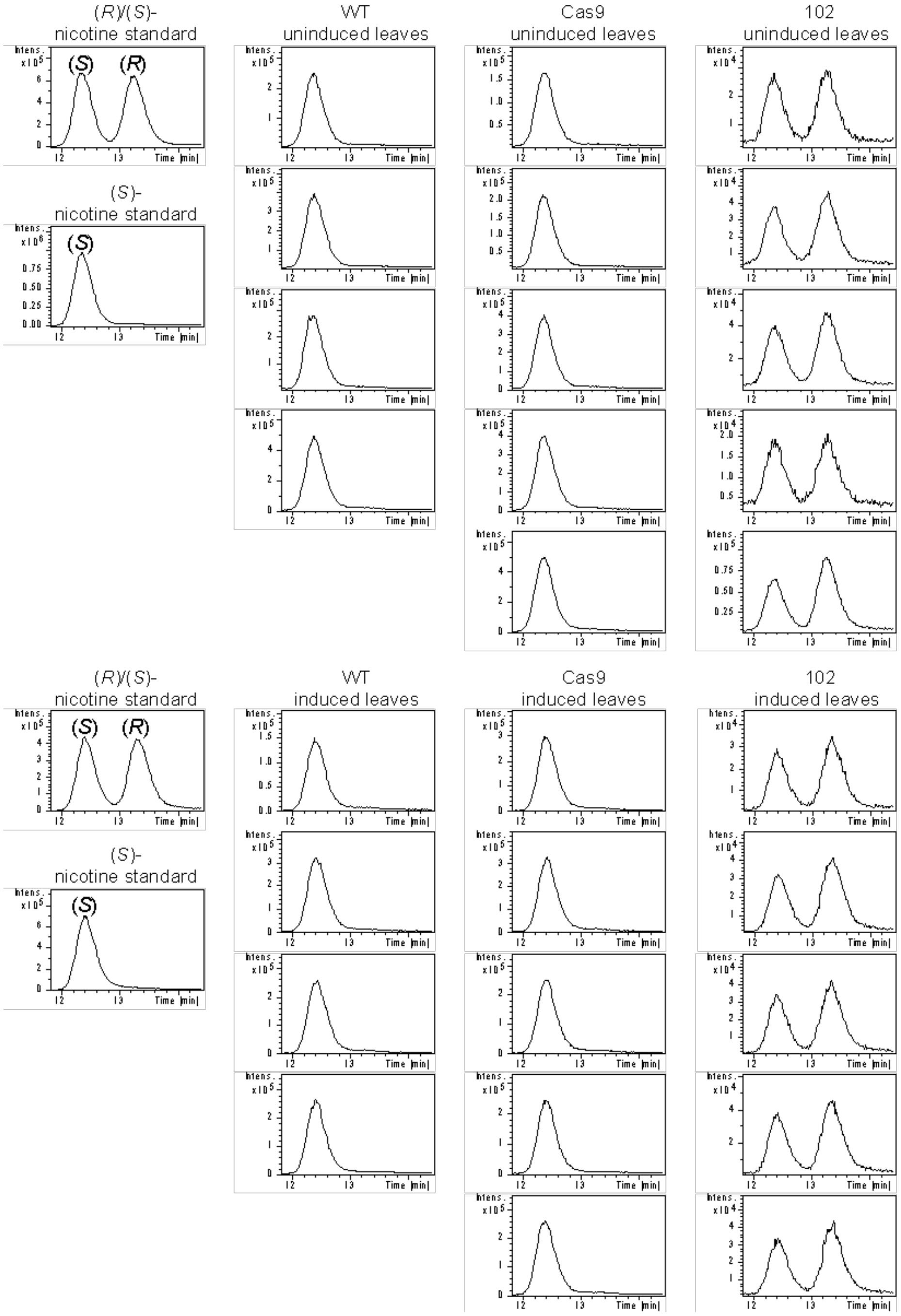

Fig. S4 Dihydrometanicotine DMN accumulation in roots of the quintuple NbBBL mutant (line 102) in comparison to two control lines (WT and Cas9), as analyzed by LC-MS. Seedlings were grown under hydroponic conditions, and roots were harvested 5 days after induction with MeJa. Traces are extracted ion chromatograms ([M+H]+) corresponding to nicotine (black) or DMN (blue). A total of five to six biological replicates per line were analyzed. The two traces at the top correspond to a (S)-nicotine standard and a DMN standard.

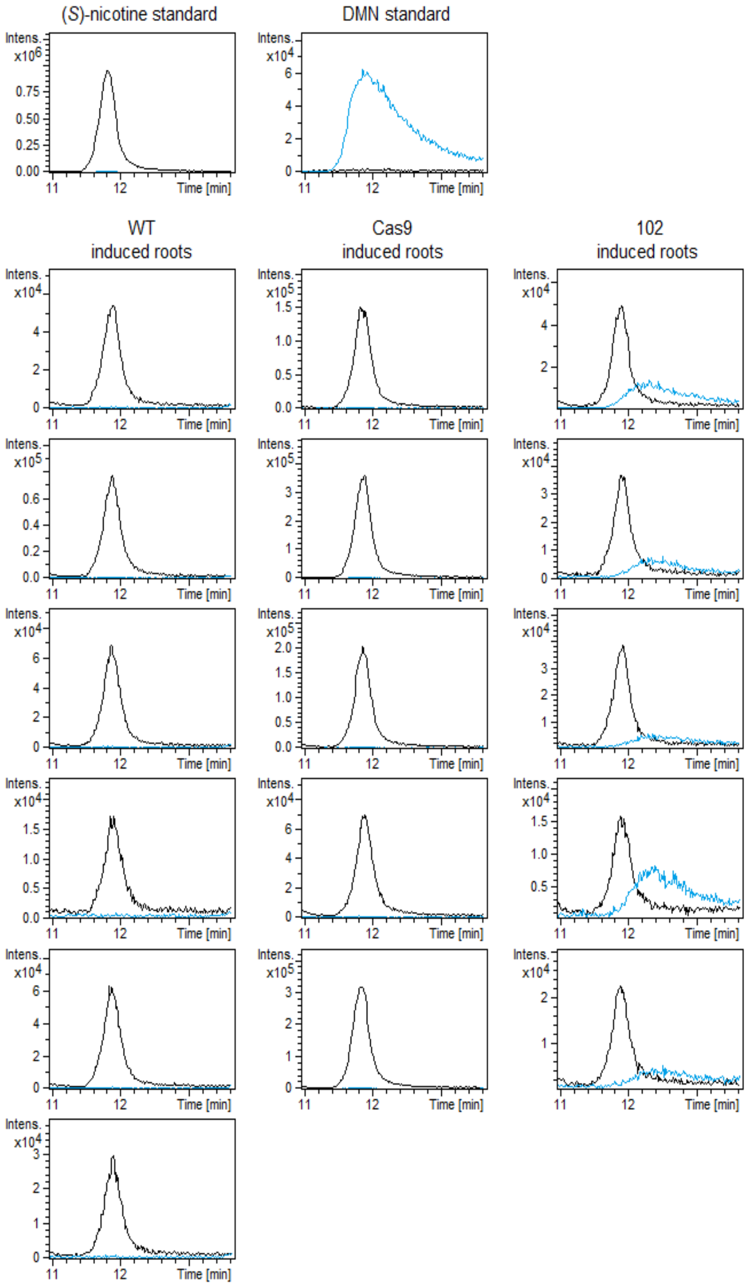

Table S1 Primers used for expression analysis of *Nicotiana benthamiana berberine-bridge like* (*NbBBL*) genes.

	RTqPCR primer (forward)	RTqPCR primer (reverse)
**NbBBLa**	CGTGGTTCAACGCAAGAGAAC	ACTAACAACGGAATCTCTCTCAAGG
**NbBBLb**	TGATCCACTAAATGTTTTCCGC	ACAACGGAATCTCTCTCAACCT
**NbBBLc**	GTTCAACGCAAGACAATAAGTATAGC	CTCGTTGAATTGGATCAATATCGC
**NbBBLd**	CCTCAATGCAAGAGCATACCTAC	CCTTTATGATCCCAGGAGGCA
**NbEF1a**	AGCTTTACCTCCCAAGTCATC	AGAACGCCTGTCAATCTTGG

Table S2 Primers used for amplification of sgRNA scaffolds. The plasmid vector pEPOR1CB0022 (Addgene#117537), which contains the sgRNA stem extension scaffold sequence first reported by Chen et al. Cell 155(7):1479–91 (2013), was used as a template. Details of the U6-promoter and L1 acceptor that the resulting amplicon was assembled with are also provided.

sgRNA #	L1 acceptor	U6 Promoter	F primer	R primer
**19**	Position 3 pICH47751 Addgene #48002	pICSL90002 (AtU6-26) Addgene #68261	tgtGGTCTCtattGTTATTAGACCGAAAAGCCAgtttaagagctatgctggaaac	tGGTCTCtagcgaaaaaaagcaccgact
**35**	Position 4 pICH47761 Addgene #48003	pICSL90002 (AtU6-26) Addgene #68261	tgtGGTCTCtattGGGAGCTTATATCAACTACTgtttaagagctatgctggaaac	

Table S3 Primers used for construction of mobile single guide RNA plasmid vectors. The plasmid vector pEPQDKN0761 (Addgene #185630) containing an sgRNA fused to truncated flowering locus T was used as a PCR template.

	F Primer	R Primer
Primers to create an amplicon encoding mbgRNA5 used to construct a vector containing one mobile guide RNA targeting BBLa/BBLb/BBLc/BBLd/BBLd' (used to create plant line 102)	aCGTCTCgcaggcacctgcaacgAAACTATGAAATCAGAGTAAGGTGgttttagagctag	tCGTCTCccgagcacctgctagtCACTttggccataagtaaccttt
Primers to create an amplicon encoding mbgRNA17 used to construct a vector containing two mobile guide RNAs targeting BBLd and BBLd' (used to create plant line 138)	aCGTCTCgcaggcacctgcaacgAAACATTGAGATTTGTTGCAGAGAgttttagagctag	tCGTCTCccgagtcacctgctagtGCACgctcaacacgtacccggccgcgattggccataagtaacctttagagt
Primers to create an amplicon encoding mbgRNA97 used to construct a vector containing two mobile guide RNAs targeting BBLd and BBLd' (used to create plant line 138)	aCGTCTCgcaggcacctgcaacgGTGCGTTTAGAGGACGCTGCGAATgttttagagctag	tCGTCTCccgagcacctgctagtCACTttggccataagtaaccttt

Table S4 Primers used for genotyping *Nicotiana benthamiana* plants with Cas9-induced mutations. Asterisks indicate a phosphorothioate bond.

	**PCR/sequencing primer (forward)**	**PCR/sequencing primer (reverse)**
**NbBBLa**	ttagatctcaaactgtatattatatatacaatgcag	ctaacaacggaatctctctcaag*g
**NbBBLb**	tattatccttctgttctcaaactgctc	ctaacaacggaatctctctcaac*c
**NbBBLc**	atgtttctactcataattctgatcagct	tttattaggaaagttaacaacgtacatttg
**NbBBLd**	ctgtgcactcttccttgtataaagc	gctgaaaatccatgaacgtc*g
**NbBBLd’**	gaagtaaacgaaagttgcaatgaa*g	aattaaagtaataggacaactaacaatatgacc
**Sequencing primer** Used to sequence amplicons of BBLa/BBLb/BBLc created using the primers above	aatttattacgccattgccaag	
	**ddPCR primer (forward)**	**ddPCR primer (reverse)**
nptII	cctgccgagaaagtatccat	tcttcgtccagatcatcctg
***Rdr1* (reference gene)**	gttacgccatccatgtgttg	cagagttcaatttgccagca

Table S5 Comparison of Nicotiana benthamiana genome assemblies. Metrics for the assemblies produced in this study (10x, 10x + ONT) and a recently published assembly (PacBio HiFI + HiC; Kurotani et al., 2023). For the merqury k-mer analysis, two k-mer databases were constructed: one from the Illumina pair-end data generated in this study and the other from the MGI pair-end data generated to estimate the genome size in Kurotani et al., 2023.

	**Assembly**		
**Statistics**	**10x**	**10x + ONT**	**PacBio HiFi + Hi-C**
**Genome size**	2,981,676,209	2,994,235,606	2,926,135,461
**%GC**	37.96%	37.96%	38.12%
**Largest contig**	33,863,123	47,606,874	184,452,736
**Number of scaffolds**	58,989	50,663	1,668
**N50 (L50)**	5,442,096 (153)	13,672,338 (68)	141,754,421 (10)
**N90 (L90)**	22,681 (3,185)	37,929 (1,810)	110,643,690 (19)
**Completeness (Illumina)**	96.53%	97.82%	99.33%
**QV (Illumina)**	52.64	37.88	46.43
**Completeness (MGI)**	96.88%	96.75%	98.16%
**QV (MGI)**	46.11	37.43	43.37
**Complete BUSCOs**	2257 (97.0%)	2270 (97.6%)	2296 (98.7%)
**Single copy BUSCOs**	865 (37.2%)	859 (36.9%)	737 (31.7%)
**Duplicated BUSCOs**	1392 (59.8%)	1411 (60.7%)	1559 (67%)
**Fragmented BUSCOs**	34 (1.5%)	28 (1.2%)	9 (0.4%)
**Missing BUSCOs**	35 (1.5%)	28 (1.2%)	21 (0.9%)

Table S6 NbBBLa genotypes in Nicotiana benthamiana plants with Cas9-mediated mutations. The target sequences for each guide RNAs (gRNAs) are provided in the top row; the protospacer adjacent motif (PAM) is underlined, bases in orange text indicate identity to NbBBLa. The sequence at the equivalent target locus in each line is shown with mutated bases/deletions in blue. For each line, the location at which the amino acid (aa) sequence first becomes out of frame is given, as well as the position of the first premature stop-codon. Not applicable (NA) indicates that a given gRNA was not included in the construct used to produce this line. Sequences corresponding to mbgRNA17 are not shown as no mutations were detected in any line.

NbBBLa				
**Plant line**	**mbgRNA5**<br>TATGAAATCAGAGTAAGGTGCGG	**sgRNA19**<br>GTTATTAGACCGAAAAGCCATGG	**sgRNA35**<br>GGGAGCTTATATCAACTACTTGG	**mbgRNA97 (rev strand)**<br>CCGATTCGCAGCGTCCTCTAAAC
**102**	**Bi-allelic**<br>TATGAAATCAGAGTAA-TGCGG<br>frameshift at aa10, stop codon at aa115<br>TATGAAATCAG-----GGTGCGG<br>frameshift at aa10, stop codon at aa114	NA	NA	NA
**138**	NA	NA	NA	**Wild type**<br>TCGATTTGCTGCATCTTACATGC
**159**	NA	**Bi-allelic**<br>GTTATTAGACCGAAAAG [illegible]______ ______________[illegible] TACTTGG<br>sequence between sgRNA19 and sgRNA35 is inverted, frameshift at aa214 and stop codon at aa222<br>GTTATTAGACCGAAAAGACCATGG [illegible]75 bp del[illegible]<br>frameshift at aa214 and stop codon at aa217		NA
**162**	NA	**Homozygous**<br>GTTATTAGACCGAAAAGACCATGG [illegible]75 bp del[illegible]<br>frameshift at aa214 and stop codon at aa217		NA
**169**	NA	**Wild type**<br>GTTATTAGACCGAAAAGCCATGG	GGGAGCTTATATCAACTACTTGG	NA
**187**	NA	**Wild type**<br>GTTATTAGACCGAAAAGCCATGG	GGGAGCTTATATCAACTACTTGG	NA
**193**	NA	**Homozygous**<br>GTTATTAGACCGAAAAGACCATGG	GGGAGCTTATATCAAC-ACTTGG<br>frameshift at aa214 and stop codon at aa217	NA
**196**	NA	**Wild type**<br>GTTATTAGACCGAAAAGCCATGG	GGGAGCTTATATCAACTACTTGG	NA
**198**	NA	**Homozygous**<br>GTTATTAGACCGAAAAGACCATGG	GGGAGCTTATATCAACTACTTGG<br>frameshift at aa214 and stop codon at aa217	NA

Table S7 NbBBLb genotypes in Nicotiana benthamiana plants with Cas9-mediated mutations. The target sequences for each guide RNAs (gRNAs) are provided in the top row; the protospacer adjacent motif (PAM) is underlined, bases in orange text indicate identity to NbBBLb. The sequence at the equivalent target locus in each line is shown with mutated bases/deletions in blue. For each line and mutation, the location at which the amino acid (aa) sequence becomes out of frame is given, as well as the position of the first premature stop-codon. Not applicable (NA) indicates that a given gRNA was not included in the construct used to produce this line. Sequences corresponding to mbgRNA17 are not shown as no mutations were detected in any line.

<table>
<tr><th colspan="5">NbBBLb</th></tr>
<tr><th>Plant line</th><th>mbgRNA5
TATGAAATCAGAGTAAGGTGCGG</th><th>sgRNA19
GTTATTAGACCGAAAAGCCATGG</th><th>sgRNA35
GGGAGCTTATATCAACTACTTGG</th><th>mbgRNA97 (rev strand)
CCGATTCGCAGCGTCCTCTAAAC</th></tr>
<tr><td>102</td><td>Homozygous
TATGAAATCAGAGTAAGGGTGCGG
frameshift at aa106 and stop codon at aa120</td><td>NA</td><td>NA</td><td>NA</td></tr>
<tr><td>138</td><td>NA</td><td>NA</td><td>NA</td><td>Wild type
tcgatttgctgcatcttacatgc</td></tr>
<tr><td>159</td><td>NA</td><td colspan="2">Homozygous
GTTATTAGACCGAAAA-CCATGG GGGAGCTTATA-------CTTGG
frameshift at aa218 and stop codon at aa252</td><td>NA</td></tr>
<tr><td>162</td><td>NA</td><td colspan="2">Homozygous
GTTATTAGACCGAAAA-CCATGG GGGAGCTTATA-------CTTGG
frameshift at aa218 and stop codon at aa252</td><td>NA</td></tr>
<tr><td>169</td><td>NA</td><td colspan="2">Homozygous
GTTATTAGACCGAAAA-CCATGG GGGAGCTTATATCAACTACTTGG
frameshift at aa218 and stop codon at aa252</td><td>NA</td></tr>
<tr><td>187</td><td>NA</td><td colspan="2">Wild type
GTTATTAGACCGAAAAGCCATGG GGGAGCTTATATCAACTACTTGG</td><td>NA</td></tr>
<tr><td>193</td><td>NA</td><td colspan="2">Homozygous
GTTATTAGACCGAAAAGCCATGG GGGAG------------ACTTGG
12 bp in frame deletion replacing 5 residues (AYINY) with a new residue (D) at aa480</td><td>NA</td></tr>
<tr><td>196</td><td>NA</td><td colspan="2">Homozygous
GTTATTAGACCGAAAAAGCCATGG GGGAGCTTATATCAACT---TGG
frameshift at aa218 and stop codon at aa250</td><td>NA</td></tr>
<tr><td>198</td><td>NA</td><td colspan="2">Homozygous
GTTATTAGACCGAAAAGACCATGG GGGAGCTTATATCAACTACTTGG
frameshift at aa218 and stop codon at aa250</td><td>NA</td></tr>
</table>

Table S8 NbBBLc genotypes in Nicotiana benthamiana plants with Cas9-mediated mutations. The target sequences for each guide RNAs (gRNAs) are provided in the top row; the protospacer adjacent motif (PAM) is underlined and bases in orange text indicate identity to NbBBLc. The sequence at the equivalent target locus in each line is shown with mutated bases/deletions in blue. For each line, the location at which the amino acid (aa) sequence first becomes out of frame is given, as well as the position of the first premature stop-codon. Not applicable (NA) indicates that a given gRNA was not included in the construct used to produce this line. Sequences corresponding to mbgRNA17 are not shown as no mutations were detected in any line.

NbBBLc				
**Plant line**	**mbgRNA5**<br>TATGAAATCAGAGTAAGGTGCGG	**sgRNA19**<br>GTTATTAGACCGAAAAGCCATGG	**sgRNA35**<br>GGGAGCTTATATCAACTACTTGG	**mbgRNA97 (rev strand)**<br>CCGATTCGCAGCGTCCTCTAAAC
**102**	**Homozygous**<br>TATGAAATCAGAG----GTGCGG<br>frameshift at aa105 and stop codon at aa125	NA	NA	NA
**138**	NA	NA	NA	**Wild type**<br>tcgatttgctgcatcttacatgc
**159**	NA	**Homozygous**<br>GTTATTAGACCGA----CCATGG<br>frameshift at aa218 and stop codon at aa237	GTTATTAGACCGA----CCATGG	NA
**162**	NA	**Homozygous**<br>GTTATTAGACCGA----CCATGG<br>frameshift at aa218 and stop codon at aa237	GTTATTAGACCGA----CCATGG	NA
**169**	NA	**Wild type**<br>GTTATTAGACCGAAAAGCCATGG	GGGAGCTTATATCAACTACTTGG	NA
**187**	NA	**Homozygous**<br>GTTATTAGACCGAAAAGCCATGG<br>frameshift at aa219 and stop codon at aa489	GGGAGCTTATA-------CTTGG	NA
**193**	NA	**Homozygous**<br>GTTATTAGACCGAAAA-CCATGG<br>frameshift at aa219 and stop codon at aa238	GGGAGCTTATA------ACTTGG	NA
**196**	NA	**Bi-allelic**<br>GTTATTAGACCGAAAAGCCATGG<br>frameshift at aa484 and stop codon at aa496<br>GTTATTAGACCGAAAAGCCATGG<br>frameshift at aa484 and stop codon at aa491	GGGAGCTTATATCAACTTACTTGG<br><br>GGGAGCTTATATCAAC-ACTTGG	NA
**198**	NA	**Wild type**<br>GTTATTAGACCGAAAAGCCATGG<br>n/a	GGGAGCTTATATCAACTACTTGG<br>n/a	NA

Table S9 NbBBLd genotypes in Nicotiana benthamiana plants with Cas9-mediated mutations. The target sequences for each guide RNAs (gRNAs) are provided in the top row; the protospacer adjacent motif (PAM) is underlined and bases in orange text indicate identity to NbBBLd. The sequence at the equivalent target locus in each line is shown with mutated bases/deletions in blue. For each line, the location at which the amino acid (aa) sequence first becomes out of frame is given, as well as the position of the first premature stop-codon. Not applicable (NA) indicates that a given gRNA was not included in the construct used to produce this line. Sequences corresponding to mbgRNA17 are not shown as no mutations were detected in any line.

NbBBLd				
**Plant line**	**mbgRNA5**<br>TATGAAATCAGAGTAAGGTGCGG	**sgRNA19**<br>GTTATTAGACCGAAAAGCCATGG	**sgRNA35**<br>GGGAGCTTATATCAACTACTTGG	**mbgRNA97 (rev strand)**<br>CCGATTCGCAGCGTCCTCTAAAC
**102**	**Homozygous**<br>TATGAAATCAGAGTAAG-TGCGG<br>frameshift at aa114 and stop codon at aa235	NA	NA	NA
**138**	NA	NA	NA	**Homozygous**<br>CCGATTACGCAGCGTCCTCTAAAC<br>frameshift at aa77 and stop codon at aa82
**159**	NA	**Wild type**<br>GCTATTAGACCGCAAAGCCATGGG	GGCAGCTTATGTCAACTATATGG	NA
**162**	NA	**Wild type**<br>GCTATTAGACCGCAAAGCCATGGG	GGCAGCTTATGTCAACTATATGG	NA
**169**	NA	**Wild type**<br>GCTATTAGACCGCAAAGCCATGGG	GGCAGCTTATGTCAACTATATGG	NA
**187**	NA	**Wild type**<br>GCTATTAGACCGCAAAGCCATGGG	GGCAGCTTATGTCAACTATATGG	NA
**193**	NA	**Wild type**<br>GCTATTAGACCGCAAAGCCATGGG	GGCAGCTTATGTCAACTATATGG	NA
**196**	NA	**Wild type**<br>GCTATTAGACCGCAAAGCCATGGG	GGCAGCTTATGTCAACTATATGG	NA
**198**	NA	**Wild type**<br>GCTATTAGACCGCAAAGCCATGGG	GGCAGCTTATGTCAACTATATGG	NA

Table S10 NbBBLd' genotypes in Nicotiana benthamiana plants with Cas9-mediated mutations. The target sequences for each guide RNAs (gRNAs) are provided in the top row; the protospacer adjacent motif (PAM) is underlined and bases in orange text indicated identity to NbBBLd'. The sequence at the equivalent target locus in each line is shown with mutated bases/deletions in blue. For each line, the location at which the amino acid (aa) sequence first becomes out of frame is given, as well as the position of the first premature stop-codon. Not applicable (NA) indicates that a given gRNA was not included in the construct used to produce this line. Sequences corresponding to mbgRNA17 are not shown as no mutations were detected in any line.

NbBBLd'				
**Plant line**	**mbgRNA5**<br>TATGAAATCAGAGTAAGGTGCGG	**sgRNA19**<br>GTTATTAGACCGAAAAGCCATGG	**sgRNA35**<br>GGGAGCTTATATCAACTACTTGG	**mbgRNA 97 (rev strand)**<br>CCGATTCGCAGCGTCCTCTAAAC
**102**	**Homozygous**<br>TATGAAATCAGAGTAAG-TGCGG<br>frameshift at aa114 and stop codon at aa238	NA	NA	NA
**138**	NA	NA	NA	**Wild type**<br>CAGATTCGCAGCGTCCTCTAATC
**159**	NA	**Wild type**<br>GCTATTAGACCGCTAAGCCATGGG	GGCAGCTTATGTCAACTATATGG	NA
**162**	NA	**Wild type**<br>GCTATTAGACCGCTAAGCCATGGG	GGCAGCTTATGTCAACTATATGG	NA
**169**	NA	**Wild type**<br>GCTATTAGACCGCTAAGCCATGGG	GGCAGCTTATGTCAACTATATGG	NA
**187**	NA	**Wild type**<br>GCTATTAGACCGCTAAGCCATGGG	GGCAGCTTATGTCAACTATATGG	NA
**193**	NA	**Wild type**<br>GCTATTAGACCGCTAAGCCATGGG	GGCAGCTTATGTCAACTATATGG	NA
**196**	NA	**Wild type**<br>GCTATTAGACCGCTAAGCCATGGG	GGCAGCTTATGTCAACTATATGG	NA
**198**	NA	**Wild type**		NA

Table S11 Adjusted *p*-values (single-step method) of ANOVA and post hoc Tukey tests for Figure 4. ***, *p*<0.001; **, *p*<0.01; *, *p*<0.05.

	**samples**	***p*-values**
**Nicotine uninduced leaves**	138 – 102	***
	159 – 102	
	162 – 102	
	169 – 102	***
	187 – 102	***
	193 – 102	
	196 – 102	***
	198 – 102	
	WT – 102	**
	TC WT – 102	
	CAS9 – 102	**
	159 – 138	***
	162 – 138	***
	169 – 138	
	169 – 138	
	187 – 138	
	193 – 138	***
	196 – 138	
	198 – 138	***
	WT – 138	
	TC WT – 138	
	CAS9 – 138	
	162 – 159	
	169 – 159	***
	187 – 159	**
	193 – 159	
	196 – 159	**
	198 – 159	
	WT – 159	*
	TC WT – 159	
	CAS9 – 159	*
	169 – 162	***
	187 – 162	**
	193 – 162	
	196 – 162	**
	198 – 162	
	WT – 162	**
	TC WT – 162	
	CAS9 – 162	*
	187 – 169	
	193 – 169	***

	196 – 169	
	198 – 169	**
	WT – 169	
	TC WT – 169	
	CAS9 – 169	
	193 – 187	***
	196 – 187	
	198 – 187	*
	WT – 187	
	TC WT – 187	
	CAS9 – 187	
	196 – 193	***
	198 – 193	
	WT – 193	***
	TC WT – 193	*
	CAS9 – 193	***
	198 – 196	*
	WT – 196	
	TC WT – 196	
	CAS9 – 196	
	WT – 198	
	TC WT – 198	
	CAS9 – 198	
	TC WT – WT	
	CAS9 – WT	
	CAS9 – TC WT	
**Nicotine induced leaves**	138 – 102	***
	159 – 102	
	162 – 102	
	169 – 102	***
	187 – 102	***
	193 – 102	
	196 – 102	***
	198 – 102	
	WT – 102	***
	TC WT – 102	***
	CAS9 – 102	***
	159 – 138	***
	162 – 138	***
	169 – 138	
	187 – 138	
	193 – 138	***
	196 – 138	
	198 – 138	***
	WT – 138	*

	TC WT – 138	
	CAS9 – 138	
	162 – 159	
	169 – 159	***
	187 – 159	***
	193 – 159	
	196 – 159	***
	198 – 159	
	WT – 159	***
	TC WT – 159	***
	CAS9 – 159	***
	169 – 162	***
	187 – 162	***
	193 – 162	
	196 – 162	***
	198 – 162	
	WT – 162	***
	TC WT – 162	***
	CAS9 – 162	***
	187 – 169	
	193 – 169	***
	196 – 169	
	198 – 169	***
	WT – 169	**
	TC WT – 169	**
	CAS9 – 169	
	193 – 187	***
	196 – 187	
	198 – 187	***
	WT – 187	
	TC WT – 187	
	CAS9 – 187	
	196 – 193	***
	198 – 193	
	WT – 193	***
	TC WT – 193	***
	CAS9 – 193	***
	198 – 196	***
	WT – 196	
	TC WT – 196	
	CAS9 – 196	
	WT – 198	***
	TC WT – 198	***
	CAS9 – 198	***
	TC WT – WT	

	CAS9 – WT	
	CAS9 – TC WT	
**Anabasine uninduced leaves**	138 – 102	***
	159 – 102	
	162 – 102	
	169 – 102	***
	187 – 102	**
	193 – 102	
	196 – 102	***
	198 – 102	*
	WT – 102	**
	TC WT – 102	
	CAS9 – 102	**
	159 – 138	***
	162 – 138	***
	169 – 138	
	187 – 138	
	193 – 138	***
	196 – 138	
	198 – 138	
	WT – 138	
	TC WT – 138	
	CAS9 – 138	
	162 – 159	
	169 – 159	***
	187 – 159	**
	193 – 159	
	196 – 159	***
	198 – 159	
	WT – 159	**
	TC WT – 159	
	CAS9 – 159	**
	169 – 162	***
	187 – 162	**
	193 – 162	
	196 – 162	***
	198 – 162	
	WT – 162	*
	TC WT – 162	
	CAS9 – 162	**
	187 – 169	
	193 – 169	***
	196 – 169	
	198 – 169	
	WT – 169	

	TC WT – 169	
	CAS9 – 169	
	193 – 187	**
	196 – 187	
	198 – 187	
	WT – 187	
	TC WT – 187	
	CAS9 – 187	
	196 – 193	***
	198 – 193	*
	WT – 193	**
	TC WT – 193	
	CAS9 – 193	***
	198 – 196	
	WT – 196	
	TC WT – 196	
	CAS9 – 196	
	WT – 198	
	TC WT – 198	
	CAS9 – 198	
	TC WT – WT	
	CAS9 – WT	
	CAS9 – TC WT	
	138 – 102	***
	159 – 102	
	162 – 102	
	169 – 102	***
	187 – 102	***
	193 – 102	
	196 – 102	***
	198 – 102	
	WT – 102	***
	TC WT – 102	***
**Anabasine induced leaves**	CAS9 – 102	***
	159 – 138	***
	162 – 138	***
	169 – 138	
	187 – 138	
	193 – 138	***
	196 – 138	
	198 – 138	***
	WT – 138	
	TC WT – 138	
	CAS9 – 138	
	162 – 159	

	169 – 159	***
	187 – 159	***
	193 – 159	
	196 – 159	***
	198 – 159	
	WT – 159	***
	TC WT – 159	***
	CAS9 – 159	***
	169 – 162	***
	187 – 162	***
	193 – 162	
	196 – 162	***
	198 – 162	
	WT – 162	***
	TC WT – 162	***
	CAS9 – 162	***
	187 – 169	
	193 – 169	***
	196 – 169	
	198 – 169	***
	WT – 169	
	TC WT – 169	
	CAS9 – 169	
	193 – 187	***
	196 – 187	
	198 – 187	***
	WT – 187	
	TC WT – 187	
	CAS9 – 187	
	196 – 193	***
	198 – 193	
	WT – 193	***
	TC WT – 193	***
	CAS9 – 193	***
	198 – 196	***
	WT – 196	
	TC WT – 196	
	CAS9 – 196	
	WT – 198	*
	TC WT – 198	
	CAS9 – 198	*
	TC WT – WT	
	CAS9 – WT	
	CAS9 – TC WT	
	169 – 138	

**Anatabine uninduced leaves**	187 – 138	
	196 – 138	
	198 – 138	
	WT – 138	
	TC WT – 138	**
	CAS9 – 138	
	187 – 169	
	196 – 169	
	198 – 169	
	WT – 169	
	TC WT – 169	
	CAS9 – 169	
	196 – 187	
	198 – 187	
	WT – 187	
	TC WT – 187	
	CAS9 – 187	
	198 – 196	
	WT – 196	
	TC WT – 196	
	CAS9 – 196	
	WT – 198	
	TC WT – 198	
	CAS9 – 198	
	TC WT – WT	
	CAS9 – WT	
	CAS9 – TC WT	
**Anatabine induced leaves**	169 – 138	
	187 – 138	
	196 – 138	
	198 – 138	***
	WT – 138	
	TC WT – 138	**
	CAS9 – 138	
	187 – 169	
	196 – 169	
	198 – 169	***
	WT – 169	
	TC WT – 169	*
	CAS9 – 169	
	196 – 187	
	198 – 187	***
	WT – 187	
	TC WT – 187	*
	CAS9 – 187	

	198 – 196	***
	WT – 196	
	TC WT – 196	*
	CAS9 – 196	
	WT – 198	***
	TC WT – 198	***
	CAS9 – 198	***
	TC WT – WT	
	CAS9 – WT	
	CAS9 – TC WT	

Table S12 Adjusted *p*-values (single-step method) of ANOVA and post hoc Tukey tests for Figure 6. ***, $p<0.001$; **, $p<0.01$; *, $p<0.05$

	samples	*p*-values
**nicotine**	Cas9 – 102	***
	WT – 102	***
	WT – Cas9	
**anabasine**	Cas9 – 102	***
	WT – 102	***
	WT – Cas9	*
**anatabine**	Cas9 – 102	***
	WT – 102	***
	WT – Cas9	

Methods S1 Genome Assembly

Production of paired-end reads

DNA was purified from young leaf tissue using a modified CTAB-based protocol (Michael *et al.*, 2018). Briefly, 5 g of frozen powdered leaf tissue was incubated with 20 mL CTAB lysis buffer (100 mM Tris-HCl, 2% CTAB, 1.4 M NaCl, 20 mM EDTA, pH 8.0) containing 20 µg/ml proteinase K for 20 minutes at 55 °C. Aliquots of 2.5 mL were extracted with 0.5 volumes of chloroform followed by an equal volume of 25:24:1 phenol:chloroform:isoamyl alcohol (PCI). DNA was precipitated from the upper aqueous phase with 0.7 volumes of ice-cold isopropanol. The pellet was washed with ice-cold 70% ethanol and air-dried at room temperature before resuspension in TE (10 mM Tris-HCl pH 7.5, 1 mM EDTA) containing 1.4 mg/ml RNase A. RNase A was then removed by two extractions with PCI and DNA was precipitated as above and resuspended in TE. Samples were pooled on a Genomic DNA Clean & Concentrator-10 column (Zymo Research, Irvine, CA, USA) and eluted in 50 µl DNA elution buffer (10 mM Tris-HCl, pH 8.5, 0.1 mM EDTA). Amplification free, Illumina compatible libraries were constructed using the Hyper Prep kit (Kapa Biosystems, Wilmington, MA, USA). DNA was sheared to 1 Kbp using an S2 column (Covaris, Woburn, MA, USA) and Kapa Pure Beads (Kapa Biosystems) were used to remove molecules of less than <500 bp. DNA molecules were end repaired, A-tailed and appropriate Illumina-compatible indexed adapters were ligated. Library QC was performed using a BioAnalyzer high sensitivity chip (Agilent, Santa Clara, CA, USA) and the concentration of viable library molecules measured using qPCR. For sequencing, libraries were loaded at 9 pM based on the qPCR concentration with an average molecule size of 625 bp. Libraries were sequenced on an Illumina HiSeq 2500 yielding 272,102,680 250 bp reads. Paired-end reads were deposited in the European Nucleotide Archive (ENA) under project number PRJEB37024 (accession numbers: ERR3971933 and ERR3971934).

Production of Oxford Nanopore Technologies (ONT) long-reads

DNA for sequencing on the MinION (Oxford Nanopore, Oxford, UK) was purified from leaf tissue sampled from an individual plant, using a modified version of the CTAB protocol above. Briefly 1.0 g of frozen powdered leaf tissue was mixed with 3 mL CTAB lysis buffer containing 20 µg/mL proteinase K and incubated at 55 °C at 125 *x g*. After 15 minutes, 20 µl of 100 mg/mL RNaseA was added and incubation continued for 15 minutes before a further 20 µL of 100 mg/mL RNaseA was added. Extractions with chloroform and PCI were performed as above except that wide-bore pipette tips were used at all stages. DNA was precipitated and resuspended in 25 µl TE. Yields (8-21 µg at 350-900 ng/µL) were determined using the Qubit fluorometer (ThermoFisher, Waltham, MA, USA). Fragments of >40 Kbp were collected for library preparation using BluePippin pulsed-field electrophoresis (Sage Science, Beverly, MA, USA). DNA was stored at 4 °C overnight before proceeding directly to library preparation. Libraries were constructed and sequenced using the Oxford Nanopore Technologies (ONT) SQK-LSK109 library construction kit and FLO-106 flow cells according to the manufacturers' instructions. A total of nine MinION flow cells were used yielding between 3 and 20 Gbp per flow cell. Two flow cells were loaded with libraries constructed from non-fragmented DNA. Two

flow cells were loaded with libraries constructed from DNA sheared to 40 Kbp using the Hydroshear (Digilab, Hopkinton, Massachusetts, USA). Molecules <25 Kbp were removed using a Blue Pippin (Sage Scientific, Massachusetts, USA) with a 0.75% cassette. Two flow cells were loaded with libraries constructed from DNA sheared to 40 Kbp and molecules of <15 Kbp removed. Three flow cells were loaded with libraries constructed from DNA sheared to 20 Kbp using a G-tube (Covaris, Woburn, Massachusetts, USA). Reads were deposited in the ENA under project number PRJEB37025, accession numbers ERR3971506-509 and ERR3972081-83.

Production of Chromium linked reads

DNA for Chromium linked-read sequencing (10X Genomics) was purified from leaf tissue sampled from an individual plant using the GE Healthcare Nucleon™ PhytoPure™ Genomic DNA Extraction Kit (Fisher Scientific, Loughborough, Leicestershire, UK) following the manufacturer's instructions except that, following precipitation with an equal volume of ice-cold isopropanol, DNA was collected by centrifugation at 1,300 x g for 10 min at 4 °C and the resulting DNA pellet washed with 1 mL 70% ethanol air-dried and resuspended in 40 µl Low TE (10 mM Tris-HCl pH 7.5, 0.1 mM EDTA). Purified DNA was quantified by Qubit (21 ng/µL) and analyzed on the Femto Pulse System (FP-1002-0275, Agilent, Santa Clara, CA, USA) with the dominant peak at 150,000 bp and 67% of the material being greater than 50 Kbp. The Chromium Controller instrument (10x Genomics, Pleasanton, California, USA) was used to produce a barcoded linked read library using 1.25 ng DNA input and the Chromium™ Genome Library Kit & Gel bead Kit v2 (120258, 10x Genomics) following the Chromium Genome Reagent Kits Version 2 User Guide (CG00043, 10x Genomics). Library yield was quantified using the Qubit dsDNA HS assay and insert size was determined using the 2100 Bioanalyzer High Sensitivity DNA chip (5067-4627, Agilent Technologies, Santa Clara, California, USA) and verified by qPCR. The library was diluted to 0.5 nM with EB in 18 µL and spiked with 1% PhiX Control v3 (Illumina) before being prepared for loading using a NovaSeq XP 2 lane kit v1.5 (20043130, Illumina). This was sequenced with 150 paired-end reads on an Illumina NovaSeq 6000 with NVCS 1.7.5 and RTA v3.4.4 on one lane of a NovaSeq S4 v1.5 flow cell with accompanying reagent cartridges (20028312, Illumina) yielding 891 Gb (2,971,099,042 reads). Sequencing data was demultiplexed and converted from base call (BCL) files to FASTQ files using the Illumina bcl2fastq2 conversion software (bcl2fastq version. 2.20.0), allowing for a one base-pair mismatch to the index sequence. Reads were deposited in the ENA under project numbers PRJEB37026 (accession number ERR3972084 and ERR3972085) and ERX10379414.

Genome Assembly

Chromium linked-reads were assembled using Supernova v2.1.1 (https://bio.tools/supernova) (Weisenfeld et al. 2017) with the reads subsampled to 56x raw coverage (--maxreads= 1136580012) as recommended by the Supernova documentation, resulting in an initial draft assembly with a N50 of 5.4 Mb. The assembly was scaffolded using ONT long-reads. To improve the quality of the scaffolding process, ONT long-reads were polished using the Illumina paired-end short reads with the tool ratatosk (v0.7.6.3; https://github.com/DecodeGenetics/Ratatosk) (Holley et al. 2021) with default options. Long read scaffolding was performed with ntLink (v1.3.4; https://github.com/bcgsc/ntLink) in gap-filling mode, with a k-mer size of 40 for mapping (--k=40), and a

requirement that at least four reads validate the joined contigs (--a=4). This process was repeated five times. Fragments of assembled chloroplast and mitochondrial genome were identified and excluded from the final assembly. Specifically, Minimap2 v2.22 (Li 2018) was used to align a reference chloroplast genome (accession number: MF577082.1) and multiple mitochondrial genomes of related *Nicotiana* species (accession numbers: MN651321.1, MN651322.1, and MN651323.1) to the scaffolded assembly. Contigs with greater than 99% identity and with comparable size to the reference chloroplast genome were removed, while all contigs that mapped to the three mitochondrial genomes and that had more than 70% of their sequence covered were considered mitochondrial content and also removed from the assembly.

To further identify and remove any remaining contamination, the FCSx contamination pipeline (v0.3.0; https://github.com/ncbi/fcs) was employed, which identified several contigs of bacterial and primate origin. These contigs were subsequently removed from the final assembly. To assess the completeness of the final assembly, (Benchmarking Universal Single-Copy Orthologs) BUSCO v5.3.2 (https://gitlab.com/ezlab/busco/-/releases#5.4.4) (Simão et al. 2015) was run with metaEuk as the aligner against the eudicots_odb10 database. Additionally, a Merqury v1.3 (Rhie et al. 2020) analysis was performed to obtain the k-mer completeness and the QV of the assembly, which provides a complementary assessment metric to the BUSCO score.

Methods S2 Liquid chromatography–mass spectrometry (LC–MS) analysis of methanolic extracts

LC-MS analysis

Methanolic extracts were analyzed via reversed-phase LC-MS on a Dionex UltiMate 3000 Quaternary Rapid Separation UHPLC+ focussed system (ThermoFisher Scientific) coupled to an ESI QTOF Compact mass spectrometer (Bruker, Bremen, Germany). Compounds were separated on a Kinetex® 1.7 µm EVO C18 100 Å column (100 x 2.1 mm, Phenomenex, Torrance, USA) applying an eluent flow rate of 0.3 ml/min and a column temperature of 40°C. Mobiles phases A and B consisted of 10 mM ammonia bicarbonate (pH 9.2) in water and acetonitrile, respectively, and the elution profile consisted of 0-1 min 2% B (constant), 1-16 min 2-25% B (linear), 16-24 min 25-65% B (linear), 24-26 min 65-100% B (linear), 26-27 min 100% B (constant), 27-27.5 min 100-2% B (linear), and 27.5-33 min 2% B (constant). Mass spectra were obtained in positive ionization mode with automatic MS/MS acquisition and using the following parameters: capillary voltage 4500 V, end plate off set 500 V, dry temperature 250°C, dry gas nitrogen flow rate 8.0 l/min, and nebulizing gas pressure 2.5 bar. MS spectra were recorded in an *m/z* range from 50-1000 Da (spectra rate: 6 Hz). Internal mass calibration was facilitated with Na-formate clusters. The injection volume was either 2 µl (induced leaf discs) or 10 µl (uninduced leaf discs, whole seedlings, and seedling roots). Data was visualized using DataAnalysis Version 4.3 (Bruker Compass DataAnalysis 4.3 (x64), Bruker Daltonik GmbH) and automated peak integration was performed with QuantAnalysis Version 4.3 (Bruker Compass DataAnalysis 4.3 (x64), Bruker Daltonik GmbH). Compounds were identified by comparison to commercial standards: anabasine [(±)-anabasine hydrochloride, Cayman Chemical, Ann Arbor, USA], anatabine [(*R*/*S*)-anatabine tartrate, Cayman Chemical, Ann Arbor, USA], caffeine (Sigma-Aldrich, St. Louis, USA), dihydrometanicotine (dihydrometanicotine dihydrochloride, Toronto Research Chemicals, Toronto Canada), and nicotine [(-)-nicotine, Fluka, Morristwon, USA]. Quantification of nicotine, anabasine and anatabine was carried out using external calibration curves.

(*R*)- and (*S*)-nicotine analysis

Chiral LC-MS analysis was performed as indicated above (LC-MS analysis) with the following modifications. Separation was achieved on a Lux® 3 µm AMP column (150 x 3.0 mm, Phenomenex, Torrance, USA) with an eluent flow rate of 0.3 ml/min and a column temperature of 40°C. Mobile phases A and B consisted of 10 mM ammonia bicarbonate (pH 9.2) in water and 2-propanol, respectively, and the elution program was isocratic at 20% B with a total run time of 20 min. Injection volumes were either 2 µl (MeJa-induced leaf discs) or 10 µl (uninduced leaf discs). Compounds were identified by comparison to commercial standards: caffeine (Sigma-Aldrich, St. Louis, USA), (-)-nicotine (Fluka, Morristwon, USA), and (±)-nicotine (Sigma-Aldrich, St. Louis, USA).

References

Kurotani K-I, Hirakawa H, Shirasawa K, Tanizawa Y, Nakamura Y, Isobe S, Notaguchi M. **2023**. Genome sequence and analysis of *Nicotiana benthamiana*, the model plant for interactions between organisms. *Plant & Cell Physiology*. **64**:248-257.

Michael TP, Jupe F, Bemm F, Motley ST, Sandoval JP, Lanz C, Loudet O, Weigel D, Ecker JR. 2018. High contiguity Arabidopsis thaliana genome assembly with a single nanopore flow cell. *Nat Commun.* **9**:541.
